# A fluorescence‐based yeast sensor for monitoring acetic acid

**DOI:** 10.1002/elsc.202000006

**Published:** 2021-01-18

**Authors:** Katja Hahne, Gerhard Rödel, Kai Ostermann

**Affiliations:** ^1^ Institute of Genetics, Faculty of Biology Technische Universität Dresden Dresden Germany; ^2^ Institute of Physiological Chemistry, Faculty of Medicine Carl Gustav Carus Technische Universität Dresden Dresden Germany

**Keywords:** acetic acid, biogas production, biosensor, *Saccharomyces cerevisiae*, whole cell sensor

## Abstract

Accumulation of acetic acid indicates an imbalance of the process due to a disturbed composition of the microorganisms. Hence, monitoring the acetic acid concentration is an important parameter to control the biogas process. Here, we describe the generation and validation of a fluorescence‐based whole cell sensor for the detection of acetic acid based on the yeast *Saccharomyces cerevisiae*. Acetic acid induces the transcription of a subset of genes. The 5´‐regulatory sequences (5´ URS) of these genes were cloned into a multicopy plasmid to drive the expression of a red fluorescent reporter gene. The 5´ URS of *YGP1*, encoding a cell wall‐related glycoprotein, led to a 20‐fold increase of fluorescence upon addition of 30 mM acetic acid to the media. We show that the system allows estimating the approximate concentration of acetic acid in condensation samples from a biogas plant. To avoid plasmid loss and increase the long‐term stability of the sensor, we integrated the reporter construct into the yeast genome and tested the suitability of spores for long‐term storage of sensor cells. Lowering the reporter gene's copy number resulted in a significant drop of the fluorescence, which can be compensated by applying a yeast pheromone‐based signal amplification system.

**Abbreviations**
5t´URS: 5t´‐upstream regulatory sequences
tRFP: turbo Red Fluorescent Protein
VFA: volatile fatty acid

## 1 INTRODUCTION

### 1.1 Whole‐cell sensors

The IUPAC (*International Union of Pure and Applied Chemistry*) defines a biosensor as a self‐contained system being able to provide specific analytical information [1]. The biological recognition element detecting a change in the environment is in spatial contact with a transducer element, which produces a measurable signal in response to the environmental change [1]. Besides their extraordinary sensitivity and specificity, a further advantage of biosensors compared to conventional analytical sensors is that they measure only bioavailable analytes [2, 3, 4].

Here, we focus on cellular biosensors (“whole‐cell sensors”). Microorganisms are particularly suitable to generate such whole‐cell sensors [3]. The robust yeast *Saccharomyces cerevisiae* (*S. cerevisiae*) can easily be genetically manipulated and exhibits a high physicochemical tolerance [10, 11, 12]. Yeast cells can be immobilized in membranes, hydrogels and pastes, e.g. for use in microfluidic platforms [13]. Very recently a microfluidic flow‐through device containing genetically modified yeast cells for the detection of diclofenac in wastewater has been reported [14]. Yeast cells have been used to sense a large variety of analytes including glucose [15, 16], copper ions [17, 18, 19], hormonally active substances [20, 21, 22], toxic substances [23, 24, 25] as well asantibiotics [26] or analgesics [27].

The aim of this study was to develop a sensor system based on yeast cells to detect acetic acid, with biogas production as a reference application. Biogas is a mixture of different gases that results from the anaerobic digestion of organic substances by various microorganisms in a humid environment [28]. The degradation can be differentiated into hydrolysis, acidogenesis, acetogenesis and methanation [29]. Acetic acid is an important intermediate and used as a process parameter in biogas plants [30, 31, 32, 33, 34, 35]. An accumulation of acetic acid reflects an imbalance between acid producers and consumers [36] and thus disturbance of the process [29].

The monitoring of acetic acid during biogas production by use of whole‐cell sensors does not require elaborate equipment and displays the bioavailability of the analyte, which is important for the microorganisms involved in the process.

**FIGURE 1:**
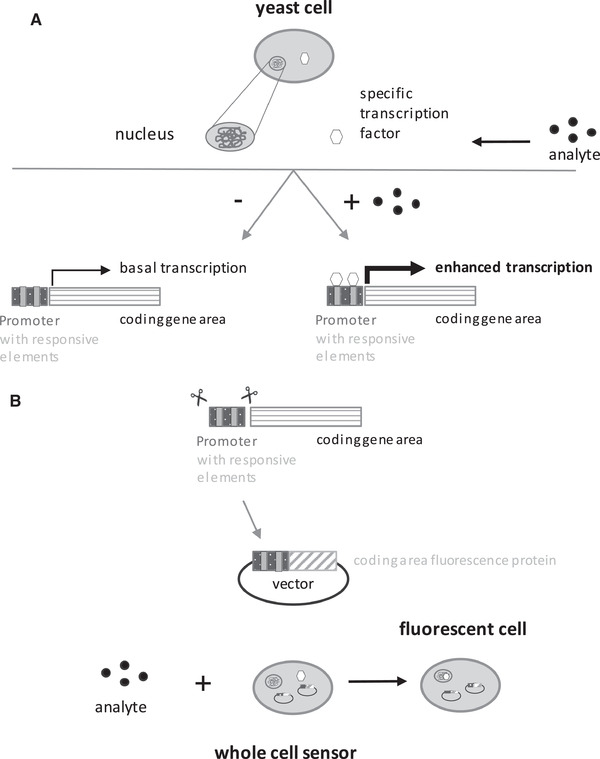
Scheme of the general strategy to generate sensor yeast cells. (A) The scheme illustrates the enhanced transcription by binding of a specific transcription factor in the promoter region of a gene. (B) shows the general strategy to generate yeast‐based whole cell sensors

Yeast cells can detect numerous substances in their environment, which often leads via binding of transcription factors to responsive elements in the 5t´ URS to the transcriptional activation of a subset of genes. Marker proteins, e.g. fluorescence proteins, are generated in the presence of the analyte, when their reading frames are combined with respective 5t´ URS, thus generating an easy to follow read‐out signal (Figure 1).

### 1.2 Acetic acid and *S. cerevisiae*

Undissociated acetic acid enters the yeast cell by diffusion via the Fps1p channel [37], where it dissociates due to the neutral cytoplasmic pH value into a proton and an acetate anion causing intracellular acidification [9, 38]. Acetic acid disturbs mitochondrial functions, interrupts the reserve metabolism and also affects the central carbon metabolism and amino acid biosynthesis [7]. Growth of yeast cells in the presence of acetic acid leads to several adaptations, e.g. in terms of construction and organization of the cell wall. The transcription factor Haa1p, which translocates from the cytosol to the nucleus in the presence of acetic acid [39], is crucial for this adaptation. It is involved in the transcriptional regulation of almost 80% of the acetic acid‐responsive genes [5]. Examination of 85 genes whose expression is Haa1p‐dependent revealed that 55% of their 5t´ URS contain at least one copy of the Haa1p‐responsive element (HRE) 5´‐(G/C)(A/C)GG(G/C)G‐3´, which is the direct target site of the transcription factor [40]. Overexpression of Haa1p has been shown to stimulate the transcription of target genes, e.g. by a factor of 30 to 40 in the case of the genes *YRO2* and *YGP1* [41].

**PRACTICAL APPLICATION**

We generated and characterized a yeast‐based whole cell sensor system to detect acetic acid, which is a critical parameter of biogas production. The system is specific for acetic acid and applicable with authentic process samples in bioreactors, e.g. in a microfluidic device. Besides for biogas production, monitoring of acetic acid may be also important for other biotechnological processes, e.g. in the food industry where acetic acid functions as a preservative and acidifier. Furthermore it is used to synthesize vinyl acetate and ethyl acetate as precursors for the chemical synthesis of polymers [5] and as a substrate for producing chemicals such as lactic acid or xylitol [6]. Moreover, acetic acid is an intermediate in bioethanol production from celluloses using yeasts [7, 8, 9].

We exploited the 5t´ URS of Haa1p‐regulated genes to create reporter gene constructs whose transcription responds to acetic acid.

### 1.3 Life cycle of *S. cerevisiae*

*S. cerevisiae* exists in three different cell types: haploid cells of either mating type a or α, and diploid cells. Each cell type is able to reproduce by mitotic division [42]. Haploid cells secrete peptide pheromones (a‐ and α‐factor, respectively), which—by binding to specific G protein‐coupled receptors on the surface of cells of the opposite mating type—activate a kinase cascade. This leads inter alia to a cell cycle arrest, morphological alterations and expression of specific mating genes [43, 44], finally resulting in the fusion of an a‐ and an α‐cell [45, 46].

Under unfavorable environmental conditions diploid cells undergo meiosis and form four haploid spores (two spores each of the mating type a and α) [47, 48, 49]. The spore wall imparts an outstanding resistance of the spores to, e.g. lytic enzymes, proteases, organic solvents, high temperature and other environmental influences [50, 51]. Therefore, spores are promising candidates as stable long‐term variants in biosensor devices, which form vegetative cells when exposed to favorable conditions.

## 2 MATERIALS AND METHODS

### 2.1 Strains

*E. coli* TOP10F´ (Invitrogen) was used for cloning and propagation of plasmids. *S. cerevisiae* strains BY4742 [MATα; *his3Δ*1; *leu2Δ*0; *lys2Δ*0; *ura3Δ*0] and BY4741 *Δbar1* [MATa; *his3Δ*1; *leu2Δ*0; *met15Δ*0; *ura3Δ*0, *YIL015w*::kanMX4] (EUROSCARF) were transformed according to Gietz and Woods (2002) [52]. Genomic integration was performed according to Mirisola et al. (2007) at the *TYR1* locus of *S.c*. BY4742 and *S.c*. BY4741 [MATa; *his3Δ*1; *leu2Δ*0; *met15Δ*0; *ura3Δ*0] (EUROSCARF) and verified by growth analysis of the transformants on media with and without tyrosine supplementation. Correct genomic integration was verified by diagnostic PCR and sequencing [53].

### 2.2 Growth media

*E. coli* was grown on LB media (1% peptone, 0.5% yeast extract, 1% NaCl). Wild type and *S. cerevisiae* cells with a genomic integrated reporter construct were cultured on YPD (2% peptone, 1% yeast extract, 2% glucose). Transformants were selected by cultivation in minimal medium (MM, 0.17% yeast nitrogen base without amino acids and with ammonium sulfate, 0.5% ammonium sulfate, 2% glucose) supplemented with the required amino acids (60 mg/L l‐histidine, 80 mg/L l‐leucine, 20 mg/L l‐methionine, 30 mg/L l‐lysine). For fluorescence measurements cells were grown in minimal medium pH 4 (MM4, pH was adjusted to 4 before autoclaving by adding 37% HCl) with the required amino acids. The measurements were started upon addition of acetic acid (Fisher Scientific GmbH, Schwerte, Germany) or volatile fatty acids to the medium in the indicated concentrations that reflect those occurring within the biogas process [54].

### 2.3 Plasmids and reporter constructs

1000 bp of the 5t´ URS of acetic acid target genes were PCR‐amplified and cloned as *Sac*I/*Spe*I fragments into the multicopy vector p426‐GPD‐tRFP (A. Groß, TU Dresden, Germany) thereby replacing the *GPD1* promoter [55, 56]. In the resulting constructs the transcription of the open reading frame (ORF) coding for tRFP (turbo Red Fluorescent Protein) is under control of the respective 5t´ URS. For integration of the reporter construct into chromosomal DNA, 1000 bp of the 5t´ URS of the respective promoters (positions ‐1 to ‐1000) were PCR‐amplified and inserted into the *Sal*I and *BamH*I sites of vector pFA6a‐GPD‐tRFP natMX6 (S. Hennig, TU Dresden, Germany), thereby replacing the *GPD1* promoter [57]. To generate the constructs for signal modulation via the yeast pheromone system, the coding sequence of the *MFα1* (Mating Factor alpha) gene was amplified and cloned as a *Spe*I/*Xho*I fragment into the vector p426‐YGP1‐tRFP replacing the *tRFP* ORF [58].

### 2.4 Fluorescence measurements

Yeast cells were cultured overnight in 50 mL Erlenmeyer flasks under shaking (180 rpm) in MM4 without acetic acid. Each well of a black 96‐well plate (BRANDplates pureGrade™ s, BRAND, Wertheim, Germany) was inoculated with 200 μL media with yeast cells at an OD_600_ of 0.1. Fluorescence measurements were performed at 30°C using the microplate reader FLUOstar Optima (BMG Labtech, Ortenberg, Germany). Fluorescence (excitation filter 550‐10, emission filter 590) and optical density (595 nm) were recorded over time. The fluorescence was determined in arbitrary units (A.U.) and normalized to OD_595_. The fluorescence induction is given as the ratio of the fluorescence signal of acetic acid treated cells to untreated cells. Three biologically independent replicas were analyzed. Mean values and standard deviations were calculated using Excel functions AVERAGE and STDEV.

### 2.5 Sporulation studies and long‐term stability

Upon mating of strains BY4741 and BY4742 with chromosomally integrated reporter constructs, diploid cells were selected on minimal media without L‐methionine and L‐lysine. Sporulation was induced by cultivation in sporulation medium (1% potassium acetate supplemented with required amino acids) for 4–5 days at 25°C. Spores were enriched by incubation of the sporulation suspension in 30% ethanol (VWR chemicals, Darmstadt, Germany) for 30 min at room temperature, followed by a 30 min incubation with zymolyase 20‐T solution (ca. 250 U/mL in 1 M sorbitol; Seikagaku Biobusiness, Tokyo, Japan) at 30°C. Spores were stored in suspension or as a pellet (at room temperature, 4°C, –20°C and –80°C) as indicated in the text.

### 2.6 Application of the yeast pheromone system for signal modulation

Erlenmeyer flasks were inoculated with overnight cultures of sensor and reporter cells in a 1:10 ratio to an OD_600_ of 1. Cultures were cultivated at 30°C under shaking (180 rpm). At the indicated time points samples were taken and placed in a black 96‐well plate. Fluorescence was determined using the microplate reader TECAN infinite M200 pro (TECAN, Männedorf, Switzerland) with excitation wavelength 553 nm, emission wavelength 587 nm, manual gain 110, number of reads 25, integration time 40 μs. Optical density was recorded at 600 nm.

## 3 RESULTS AND DISCUSSION

### 3.1 Plasmid‐based sensor for acetic acid detection

Based on available literature and data bases, candidate genes were identified, whose transcription is modulated in dependence of acetic acid: *PHM8*, *SPI1*, *TOS3*, *TPO2*, *TPO3*, *YGP1* and *YRO2* (Table 1). Except for *YRO2*, which seems to be indirectly activated by Haa1p [40, 59], all these genes possess at least one HRE in their 5t´ URS and are probably direct targets of Haa1p [40]. To generate a sensor for acetic acid detection, 1000 bp of the 5t´ URS of the candidate genes were fused to the ORF encoding tRFP and cloned it into plasmids as outlined in materials and methods. The resulting reporter constructs were transformed into *S.c*. BY4742 yielding a plasmid‐based yeast whole cell sensor to detect acetic acid (Figure 1).

**TABLE 1:** Selected yeast genes with modulated transcription in response to acetic acid

Gene	Location (1) and function (2) of the protein	References
*PHM8*	(1) Cytosol (2) Phosphatase involved in regulation of phosphatase metabolism	Reddy et al. 2008 [60]
*SPI1*	(1) Cell wall (2) Glycosylphosphatidylinositol anchored protein participating in acid‐induced remodeling of the cell wall	Simoes et al. 2006 [61]
*TOS3*	(1) Cytosol (2) Kinase taking part in adaptation to glucose limitation	Casamayor et al. 2012 [62]
*TPO2* *TPO3*	(1) Plasma membrane (2) Export of acetate anions	Mira et al. 2010 [5]
*YGP1*	(1) Cell wall (2) Glycoprotein involved in acid‐induced remodeling of the cell wall	Destruelle et al. 1994 [63]
*YRO2*	(1) Plasma membrane (2) Unknown function	Takabatake et al. 2015 [59]

**FIGURE 2:**
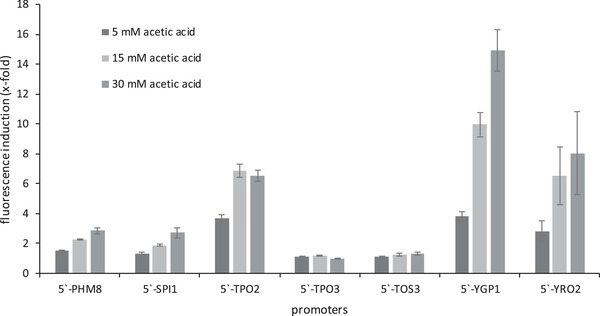
Fluorescence induction by acetic acid through different promoters. Values are standardized to an OD_600_ of 1. Mean and standard deviation of three biologically independent replicas are shown. A 96‐well plate was equipped with 200 μL media per well, without or with 5, 15 or 30 mM of acetic acid. The strains *S.c*. BY4742 + p426‐promoterX‐tRFP (X represents the respective promoter) were inoculated at OD_600_ 0.1 and analyzed at 30°C in the plate reader FLUOstar Optima. Fluorescence (550/590 nm) and optical density (595 nm) were recorded. The ratio of fluorescence of acetic acid‐treated cells to untreated control cells after 12 h of incubation is shown

In initial experiments the fluorescence signals of the transformants in the presence or absence of acetic acid in minimal medium with different pH values were determined (see Figure S1). Regarding the signal intensity, cells harbouring the reporter construct with the 5t´ URS of *YGP1* were most promising (Figure 2).

**FIGURE 3:**
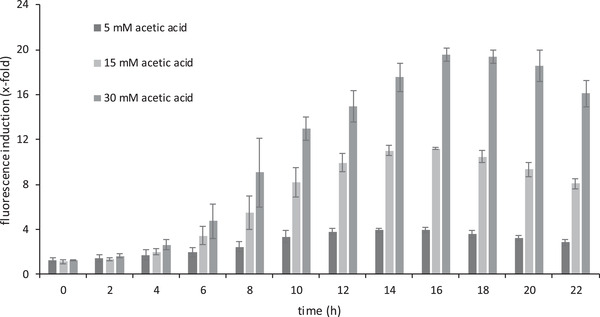
Acetic acid‐induced fluorescence induction. All values are standardized to an OD_600_ of 1. Mean and standard deviations of three biologically independent replicas are shown. (A) 96‐well plate was equipped with 200 μL media per well, without or with 5, 15 or 30 mM of acetic acid. The strain *S.c*. BY4742 + p426‐YGP1‐tRFP was inoculated with an OD_600_ 0.1 and analyzed at 30°C in the plate reader FLUOstar Optima. Fluorescence (550/590 nm) and optical density (595 nm) were recorded over a period of 22 h. The ratio of fluorescence of acetic acid‐treated cells to untreated control cells is presented

**FIGURE 4:**
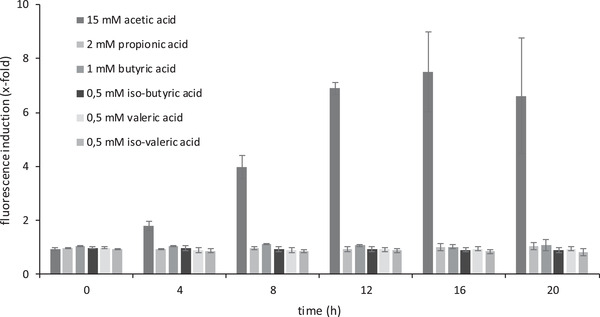
Fluorescence induction of the whole cell sensor in response to different volatile fatty acids using the plasmid‐based whole cell sensor *S.c*. BY4742 + p426‐YGP1‐tRFP. Ratio of fluorescence of acid‐treated cells to untreated control cells is displayed. The concentrations of acids represent real biogas conditions. All values are standardized to an OD_600_ of 1. Mean and standard deviations of three biologically independent replicas are shown

Therefore we focused on analyzing the growth behavior and the fluorescence signal of respective sensor cells in more detail, including their dependence on different concentrations of acetic acid (Figure 3), their cross‐sensitivity against other chemical components of the biogas process (Figure 4) and their regeneration capacity (see Figure S2).

Fluorescence was recorded in the presence of up to 30 mM acetic acid (Figure 3), which is in the range of concentrations observed in the biogas process [32, 64].

In the presence of 5 mM acetic acid, a maximum fluorescence of 600 A.U. was reached after 16 h. Using 15 mM acetic acid we observed a continuous increase of the fluorescence signal up to 1800 A.U. after 16 h, while in the presence of 30 mM acetic acid a maximum fluorescence of 3500 A.U. was observed after 20 h. Cells growing in the absence of acetic acid displayed no increase of fluorescence. The induction rates (ratio of the fluorescence of cells treated with acetic acid to that of untreated control cells) reached a maximum of 4 with 5 mM, of 11 with 15 mM, and of 19.5 with 30 mM acetic acid (Figure 3).

Despite the delay of the signal, it is sufficient to monitor a malfunction of the biogas reactor that starts with increased concentrations of volatile fatty acids (VFA, acetic acid first) lasting for days before the whole process will be tipped over. Ahring et al. (1995) monitored 2 days after introducing a disorder an increase of VFA [65]. Marchaim and Krause (1993) observed an increase acetic acid concentration on day 33 after overloading the reactor by raising the feeding of glucose on day 31 [66]. Other authors detect an accumulation of acetate 1 day after introducing a perturbation to the reactor [67].

Next, we tested the specificity of the sensor cells for acetic acid by applying other VFA, which are components in the biogas process [31, 32, 64, 68]. Addition of propionic acid, butyric acid or valeric acid to the media in concentrations that are typically occurring in the fermentation process, yielded no significant increase of the fluorescence signal (Figure 4), while 15 mM acetic acid resulted in the expected continuous increase of the signal. These data document the specificity of the sensor system for acetic acid. VFA concentrations in biogas and their relationship to each other are strongly dependent on various factors, e.g. substrate, organic loading rate, hydraulic retention time, existence of inhibitory substances, pH and temperature [35, 69, 70]. According to Franke‐Whittle et al. (2014) it is even impossible to define exact VFA concentrations as indictors for the state of biogas process, because different systems possess different “normal” [71]. Our tested VFA concentrations were based on the experience of biogas experts.

The sensor cells can be used repeatedly as shown by the fact that—in line with the presence or absence of acetic acid—the fluorescence increases and decreases. However, the response of the cells to the repeated stimulus on day 2 was significantly weaker and delayed compared to the original induction (see Figure S2). Regeneration of the fluorescence signal was investigated by two parallel cultures: In one approach sensor cells were alternately cultivated in medium with or without acetic acid, in the second approach plasmid‐based sensor cells were continuously cultivated in medium without acetic acid. While the fluorescence signal of these cells remained stable at low level during the time, the signal of the cells pre‐cultivated in medium with acid decreased during the first day of cultivation in medium without acetic acid. After adding acetic acid at day 2 it increased again over time but did not reach the level from the start (see Figure S2). All in all, our first experiments indicate that the amplitude of the signal drops over time.

### 3.2 Integration of the reporter construct into the chromosomal DNA of sensor cells

Many factors including the construct structure, plasmid copy number, expression level of target genes, cultivation media, analyte solution and physical growth parameters influence plasmid stability in yeast cells [72]. In order to increase the robustness and reliability, we integrated the reporter construct into the genome of sensor cells [53]. Chromosomally integrated sensor constructs are known for higher uniformity in fluorescence and lower cell‐to‐cell variance compared to plasmid‐based sensor versions [56].

Chromosomal integration of the *tRFP* gene under the control of the 5t´ URS of *YGP1* was performed as described in Section 2. In the presence of 15 mM acetic acid, the respective transformants exhibit an induction of fluorescence after 12 h of up to 2.5‐fold. This increase is significantly lower compared to the plasmid‐based sensor cells (8.3‐fold; see Figure S3), however, a clear and reproducible dosage dependence on acetic acid was obtained.

Next the concentration of acetic acid was determined in original samples from biogas plants. To this end, we investigated the condensate from the hydrolysis step in biogas production. Incubation of the sensor cells in undiluted condensate led to a delayed growth compared to cells grown in minimal medium (see Figure S4A) but we were able to detect acetic acid added in concentrations of 5, 15 and 30 mM to a 1:2 diluted condensate (see Figure S4B).

In order to test whether the sensor system allows for the determination of acetic acid concentrations relevant for biogas production, we monitored the fluorescence of sensor cells after incubating them for 12 h in minimal medium with decreasing amounts of acetic acid up to 30 mM. We observe a linear relationship between the fluorescence intensity and the acetic acid concentration. By using the resulting calibration curve (see Figure S4C), it is therefore possible to determine an approximate concentration of acetic acid in a condensate sample.

### 3.3 Long‐term stability of sensor cells with a chromosomal reporter construct

Robustness and long‐term stability are crucial for the application of whole cell sensors. Spores of yeast cells are extremely robust and hence of interest for long‐term use and storage of sensor cells. Therefore, derivatives of *S.c*. BY4742 and *S.c*. BY4741, both with a chromosomal integration of the *tRFP* gene under the control of the 5´ URS of *YGP1* at the *TYR1* locus, were crossed, diploid cells selected, and sporulation initiated (see materials and methods). The mixed population of sporulated and non‐sporulated cells was treated with ethanol and zymolyase solution to get rid of unsporulated cells. Spores were stored either suspended in phosphate buffered solution or as cell pellets at different temperatures. Following germination by incubation of spores on YPD media, the fluorescence signal of the vegetative cell population was determined. In line with the results shown above we observed an approximately three‐fold induction of fluorescence after 12 h incubation in the presence of 15 mM of acetic acid (see Figure S5). Similar results were obtained after storage of the spores up to 6 months at room temperature or at +4°C, –20°C or –80°C (see Figure S5). These results show that spores can provide long term stability of the sensor system.

### 3.4 Signal amplification utilizing the *S. cerevisiae* pheromone system

**FIGURE 5:**
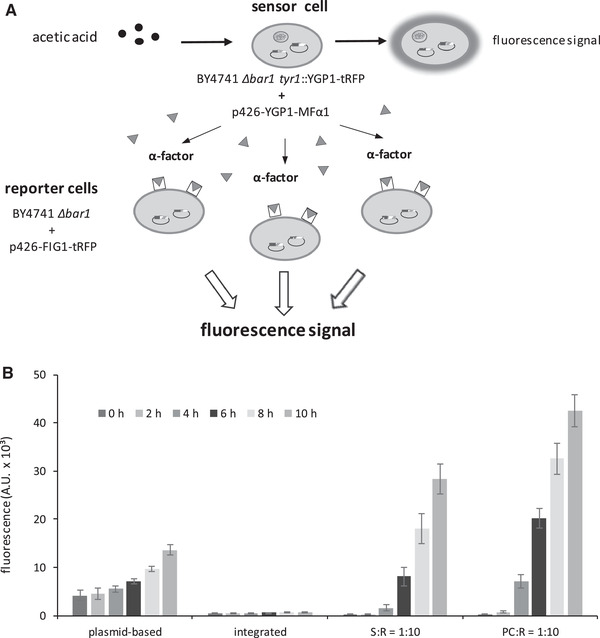
(A) Scheme for using a yeast pheromone‐based system for signal amplification. Sensor and reporter cells are co‐cultivated in a 1:10 ratio. Sensor cells with chromosomally integrated 5`‐YGP1‐tRFP are carrying the plasmid p426‐YGP1‐MFα1. Reporter cells were transformed with the plasmid p426‐FIG1‐tRFP. Sensor cells detect acetic acid and release α‐factor molecules. Besides there is a weak fluorescence signal caused by the integrated reporter gene construct. Receptor proteins on the surface of reporter cells bind the α‐factor and initiate the yeast pheromone signal cascade resulting in the induction of the FIG1 promoter and red fluorescence signal. (B) Effect of the yeast pheromone‐based signal amplification systems on fluorescence signals. Fluorescence signals were induced by adding 15 mM of acetic acid to the medium. The plasmid‐based biosensor (plasmid‐based) contains p426‐YGP1‐tRFP and the integrated biosensor (integrated) one copy of the construct 5‐YGP1‐tRFP within the TYR1 locus. Reporter cells (R) of the strain *S.c*. BY4741 *Δbar1* harboured plasmid p426‐FIG1‐tRFP. Sensor cells (S) with chromosomally integrated construct 5`‐YGP1‐tRFP were transformed with plasmid p426‐YGP1‐MFα1. *S.c*. BY4741 *Δbar1* yeast cells transformed with p426‐GPD‐tRFP functioned as positive control (PC). Cells were co‐cultivated in a 1:10 ratio. All values are standardized to an OD_600_ of 1. Mean value and standard deviations of three biologically independent replicas are shown

As outlined above, the fluorescence signal of sensor cells with a chromosomally integrated reporter construct is weaker than that of cells with a plasmid‐borne construct. In order to increase the signal intensity we applied the recently developed signal amplification system [58] based on the following principle: Upon detection of an analyte sensor cells (S) release α‐factor, which binds to a receptor on the surface of a reporter (R) a‐cell thereby activating the pheromone response pathway. In R‐cells expression of a fluorescent protein is controlled by the 5t´ URS of the *FIG1* gene, an early target of the pheromone response. Its transcription is induced approximately 100‐fold [46, 73] 20 min after activation of the pheromone signal cascade. Consequently, the R‐cells generate a strong fluorescence signal and co‐cultivation of S‐ and R‐cells is an appropriate way to intensify this signal in a yeast‐based biosensor system (Figure 5A).

In order to avoid degradation of the α‐factor through the protease activity of the Bar1 protein [74], yeast strains lacking the *BAR1* gene (*Δbar1*) were used. S‐cells were generated by transformation of strain BY4741*Δbar1* that harbours the construct 5´‐*YGP1‐tRFP* either on a plasmid or integrated in the chromosome, with a plasmid encoding the *MFα1* gene under the control of the *YGP1* 5t´ URS. In the presence of acetic acid, the transformants express tRFP and concomitantly secretes α‐factor into the medium.

Strain BY4741 *Δbar1* transformed with plasmid p426‐FIG1‐tRFP served as R‐cells, in which transcription of the *tRFP* gene is controlled by the 5t´ URS of the *FIG1* gene and hence activated by binding of α‐factor to the respective cell surface receptor [46, 73]. BY4741 *Δbar1* transformants, in which *MFα1* is under the control of the constitutive *GPD* promoter served as positive control (PC). In all co‐cultivation experiments, the ratio of sensor to reporter cells was 1:10 (Figure 5B).

When PC sensor cells were co‐cultivated with the R‐cells in 15 mM acetic acid, a maximum fluorescence signal of 43,000 A.U. was observed after 10 h (Figure 5B). Co‐cultivation of S‐ cells (BY4741 *Δbar1* *tyr1*::YGP1‐tRFP + p426‐YGP1‐MFα1) with the R‐cells yielded a fluorescence signal of 28,000 after 10 h. The effect of the signal amplification system is demonstrated by comparing the results of plasmid‐based (*S.c*. BY4741 *Δbar1* + p426‐YGP1‐tRFP) and chromosomally integrated sensor cells without the amplification system (BY4741 *Δbar1* *tyr1*::YGP1‐tRFP) under the same conditions. Here the fluorescence intensities amounted only to 14,000 and 700 A.U., respectively (Figure 5B).

## 4 CONCLUDING REMARKS

We report on the development of a yeast‐based sensor system to detect acetic acid, using biogas production as a reference application. The gene encoding tRFP was combined with the *HAA1* promoter leading to acetic‐acid inducible expression of the fluorescent protein in a concentration‐dependent manner [75].

For future applications it is might be desirable to shorten response time. In initial experiments with strains bearing deletions of genes for acetic acid importers and acetate exporters no significant influence on the response time could be observed (see Figure S6). Currently an alternative strategy with strains that overexpress the transcription factor Haa1p [8, 41] is tested.

The generated whole cell sensor measures the bioavailable acetic acid in the medium. As only undissociated acid can freely diffuse into the cell via the plasma membrane [39], the pH of the medium, which determines the degree of dissociation of the acid, plays an important role. The pH value of the medium by itself does not trigger fluorescence: even at low pH values no fluorescence signal is detectable without the addition of acetic acid (see Figure S1).

Date and colleagues (2007) already described the use of spores of *Bacillus subtilis* for long‐term preservation, storage and transport of biosensors [76]. Using spores of the sensor yeast cells we could show that storage under different conditions and temperatures of up to 6 months did not affect the capacity of the cells to generate fluorescence signals.

Genomic integration of the reporter construct results in a decreased fluorescence signal. This can be overcome by implementing the yeast pheromone system for signal amplification: Co‐cultivation of S‐ and R‐cells increased the fluorescence signal up to 40‐fold. The application of such a sensor system in a microfluidic system was recently successfully demonstrated [14]. As exploiting the yeast pheromone system leads to a cell‐cycle arrest, which in turn may modulate the fluorescence signal, the use of strains with appropriate mutations to circumvent this arrest (e.g. *far1* [77]) might be of interest.

In summary, we report the successful generation of a yeast‐based whole‐cell sensor that can detect acetic acid in a concentration‐dependent manner. The detection is specific and shows no cross‐reactivity to other VFA occurring in the biogas process. By combining with a yeast pheromone‐based amplification system, the fluorescence signal can be increased up to 40‐fold allowing the usage of yeast sensor cells with stably chromosomally integrated reported gene constructs.

## CONFLICT OF INTEREST

The authors have declared no conflict of interest.

## Supporting information

Supporting information

## Data Availability

The data that supports the findings of this study are available in the supplementary material of this article.
