# Supplementary material for: A fluorescence‐based yeast sensor for monitoring acetic acid
Source: Eng Life Sci. 2021 Jan 18;21(5):303–13. doi: 10.1002/elsc.202000006 (PMC8092980; doi:10.1002/elsc.202000006)
Supplement: Supplementary file 1 — Supporting information [file ELSC-21-303-s001.pdf]

Supporting information

1. Supplementary figures

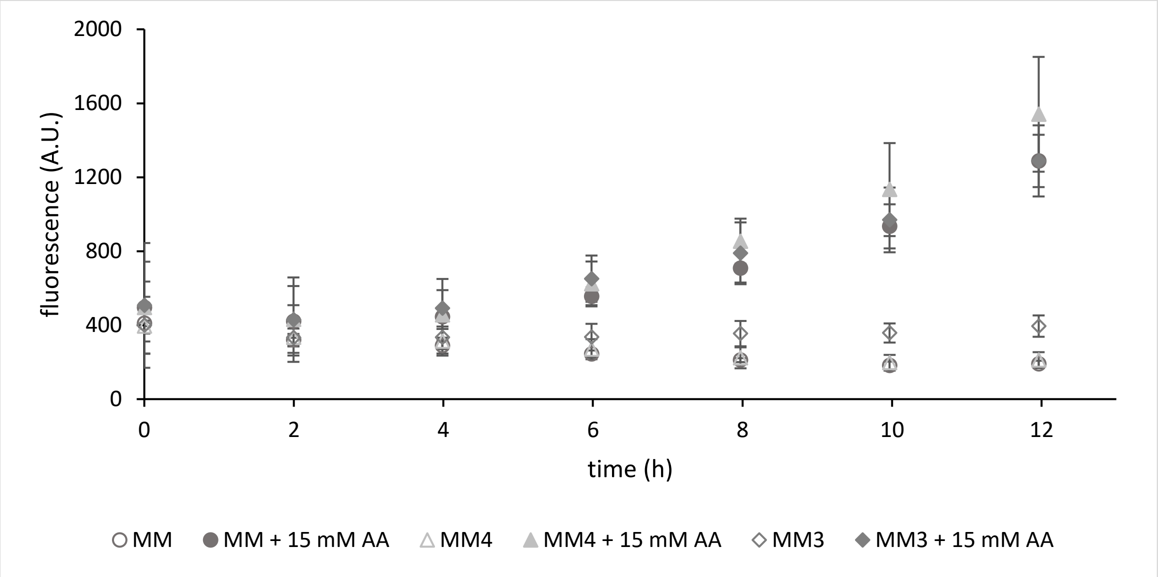

Suppl. Figure 1: **Acetic acid-induced fluorescence induction**. All values are standardized to an OD_{600} of 1. Mean and standard deviations of three biologically independent replicas are shown. A 96-well plate was equipped with 200 µl minimal media per well, without or with 15 mM of acetic acid. The strain *S.c.* BY4741 + p426-YGP1-tRFP was inoculated with an OD_{600} 0.1 and analyzed at 30 °C in the plate reader FLUOstar Optima. Fluorescence (550/590 nm) and optical density (595 nm) were recorded over a period of 12 h. Fluorescence signals in arbitrary units (A.U.) of sensor cells cultivated in different types of minimal medium are shown. MM: Minimal media pH 5, MM4: Minimal media pH 4, MM3: Minimal media pH 3.

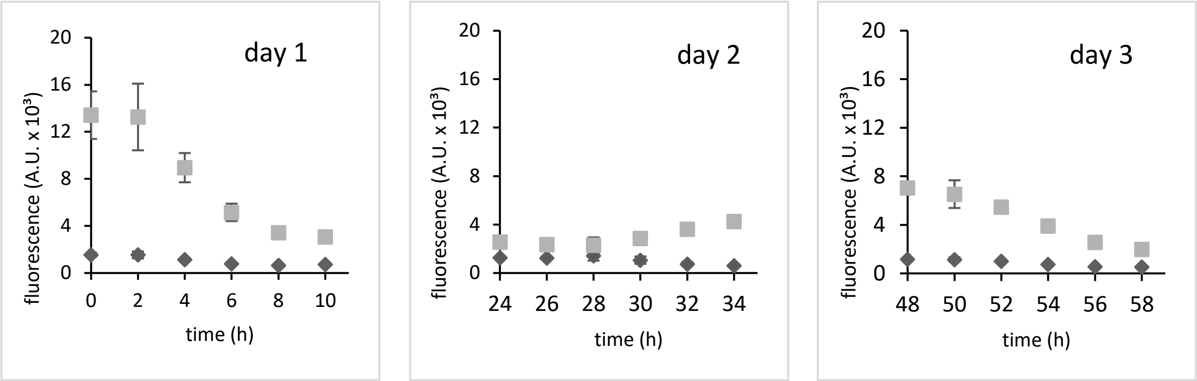

Suppl. Figure 2: Regeneration properties of the fluorescence signal of the plasmid-based whole cell sensor *S.c.* BY4742 + p426-YGP1-tRFP by alternating incubation in media containing acetic acid. Yeast cells were incubated in flasks at 30 °C. Transformed yeasts were pre-grown in medium containing 15 mM of acetic acid (□). At the beginning of day 1, 2 and 3 after measuring OD_{600}, 250 ml flasks were inoculated with cell suspension to OD_{600} of 0,5. At day 1 and 3 cells were cultivated without adding acetic acid. At day 2, 15 mM acetic acid was added to the medium right after harvesting, washing, and re-inoculating the cells. Yeast cells without adding acetic acid at any time served as control (♦). Samples were taken at indicated time points measuring growth (600 nm) and fluorescence (553/587 nm) using the TECAN infinite M200 pro. Mean and standard deviations of three biologically independent replicas are shown.

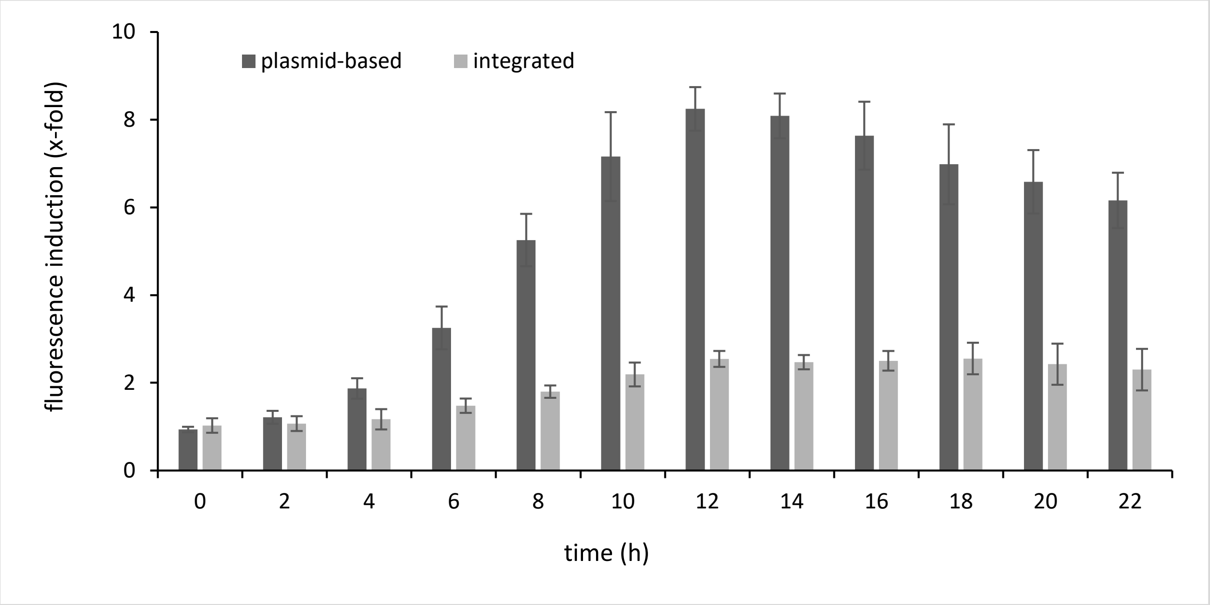

Suppl. Figure 3: Comparison of the fluorescence induction of a plasmid-based whole cell sensor (*S.c.* BY4742 + p426-YGP1-tRFP) and sensor cells carrying an chromosomally integrated construct (*S.c.* BY4742 tyr1::YGP1-tRFP) utilizing 15 mM of acetic acid. The induction presents the ratio of fluorescence of acetic acid treated cells to untreated control cells. All values are standardized to OD_{600} of 1. Mean and standard deviations of three biologically independent replicas are displayed.

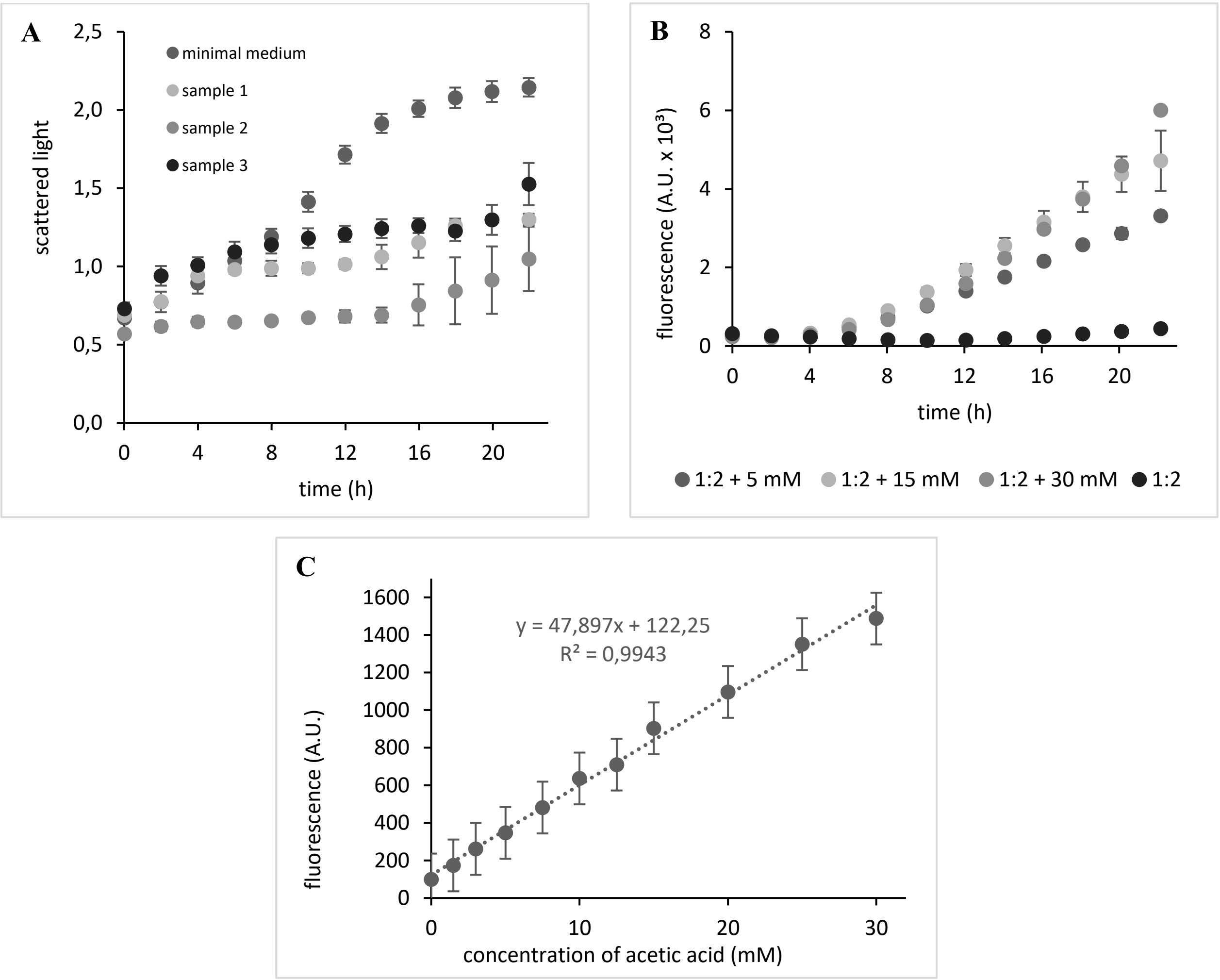

Suppl. Figure 4: Examination of condensate samples using the plasmid based whole-cell sensor *S.c.* BY4742 + p426-YGP1-tRFP. Mean and standard deviations of three biologically independent replicas are shown. A 96-well plate was inoculated with an OD_{600} 0.1 and analyzed at 30 °C in the plate reader FLUOstar Optima. Fluorescence (550/590 nm) and the optical density (595 nm) were recorded over a period of 22 h. (**A**) Growth of the whole-cell sensor incubated in different condensate samples compared to cultivation in minimal medium is shown. (**B**) Fluorescence signals in arbitrary units (A.U.) after adding acetic acid in different concentrations to a 1:2 diluted condensate are displayed. (**C**) Fluorescence values plotted against the known concentration of acetic acid at t = 12 h of incubation. The calibration curve to determine the acetic acid concentration of biogas plant samples is shown.

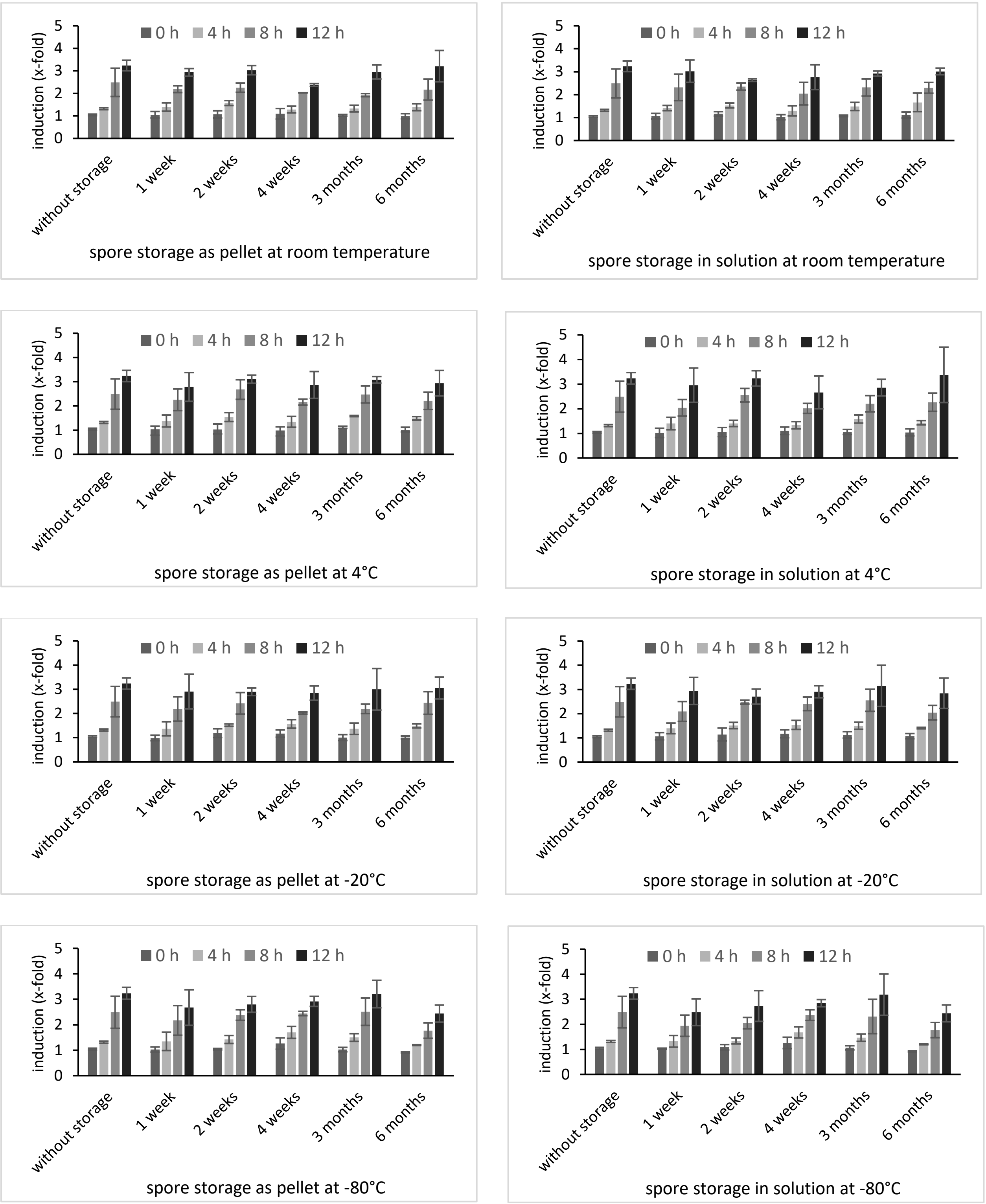

Suppl. Figure 5: Induction of fluorescence after reactivation of spores. Sensor cells containing chromosomal integrated *tRFP* were sporulated and one half of these spores were stored at room temperature as pellets (left) and the other half in phosphate-buffered solution (right) for the indicated periods. Following the spore germination, the fluorescence of the resulting vegetative cells was analyzed using FLUOstar Optima. The term "without" refers to the first measurement after generating and immediate reactivation, without any storage. The fluorescence ratio of cells treated with 15 mM acetic acid to untreated control cells is displayed. All values are standardized to OD_{600} of 1. Mean and standard deviations of three biologically independent replicas are shown.

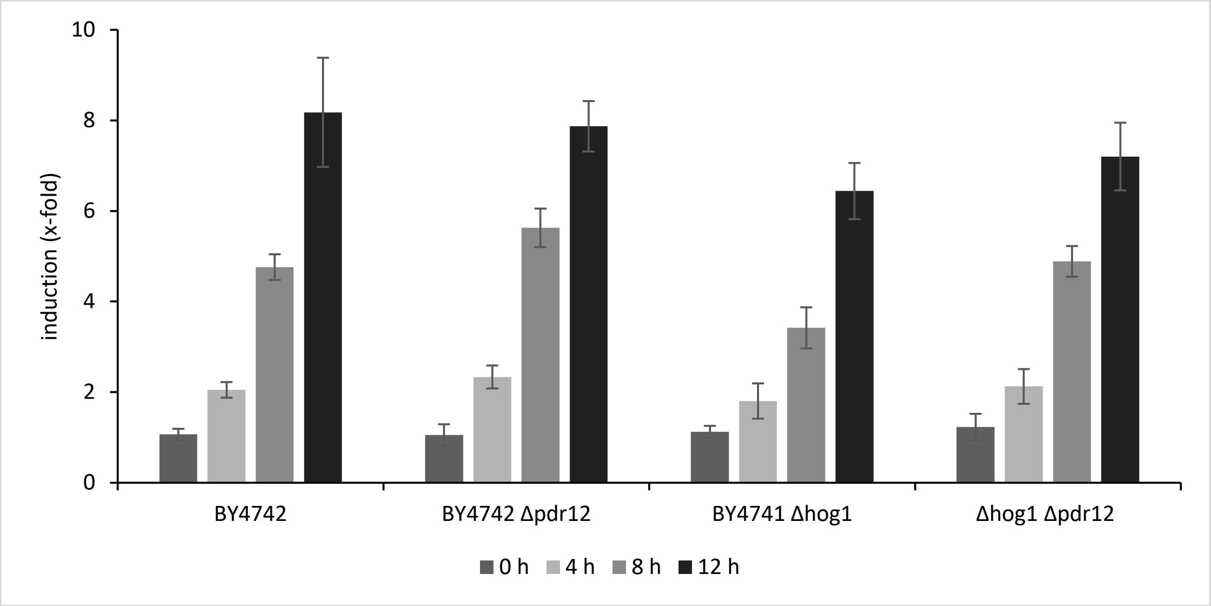

Suppl. Figure 6: Fluorescence induction of different deletion strains harbouring p426-YGP1-tRFP The induction was introduced by adding 15 mM of acetic acid. It presents the ratio of fluorescence of acetic acid treated cells to untreated control cells. All values are standardized to OD_{600} of 1. Mean and standard deviations of three biologically independent replicas are displayed.

2. DNA sequences

The 5´ URS of acetic acid target genes used for the generation of reporter gene constructs are given below. The promoter length was set to 1.000 bp. If there was another coding gene area in this part, the 5´- regulatory area was shortened accordingly. The minimal HRE motif 5´-(G/C)(A/C)GG(G/C)G-3` according to Mira *et al*. (2011) [1] is highlighted in yellow.

PHM8 **(1.000 bp)**

```
  1 AATTCTAAAC CAGAATCTTG GATCAAAACT TTACCGTGGA ACAGTAAAGA AACAGAAGAT
 61 AGCTTGATAT CACGGGAAGT CTCCAATGAA CTCAAAACAC CAGTGACAAC ACGATCTGAA
121 AGACCATCCT TATCTTGTTG CAACTTCATC ATCTTGATTT CTCTAGCGGC TGCATCTACC
181 TCCGATTCTT CAGCAGCAGC CTCCTTTTTT CTACCTAGCT TTCTGATGGT TTTACCGGCA
241 GCGGCTTTCT TAGCCTCTCT TTCAGCTTTC TTAGCATCTC TCTTGGCCTT TGACGCAGAT
301 ACTGGTGGCA TATTTGGTTC TGTCAAGTTA CTGTTTGTAG CACTGAGGGG AAGAAAACAA
361 TTTTTGCAAG AAAAAAGTTA AGAAAGAAGA AGAAAAACTA CACGGGCACT AACATGTTAA
421 ATATGATAAT ATTTTTTTTT ATAAGAGAAT CACTACCAAG TTACCTGAAC TACGTCAAGG
481 AAAAGCCATT AATTGAGATG AGGTGAACGA AGAAAAAAAA AAAGAAAAAA ATTTTTATTT
541 TTTTTTCCAT CGGTCTCCCC CCTCACCTTT CTTTTTTCAT TCGTTTAAAG TGAGATTAGG
601 GTAATGCATT TCGTATTTAG TGAGTAAACG TCAAAGGTGA TATAGGGGGT TTTTCTTTTT
661 CCAGAAAAAG AAAGAAAAAA GCCGGCGGTC GCCGCCGGAG ATGGTTGAGA AAAAAAGGGG
721 GCAGCAACGT GCTTCACATA CAAACCAAGT TCTTGGATCT GTACGATCGT TTCTGCGGGT
781 TTTTCCGTAT TTGCCGCAAT ACTGAAATGG CTCATTATTA GGAGATGTGC ATATAATGTA
841 TATACATACA TTTAGCATAT GTAATACATT CGTACATGTA TAGAAATATA TGTTTATATA
901 TATATATATA TATATATGTA TATACGAACT CGGTTTAACG CATAGTTGGT AAAAGATTTA
961 ATGTAGATAA AAAACACATA AGTTTTATAC GCACGTAATC
```

SPI1 **(923 bp)**

```
  1 TGTTTCAATT CATCGTATTT CAATGGATAC TCTTCGTATC GTTCTGCACT AAAAAGCAAA
 61 GGGTTCGCTC TTCTCGAGAG CTCTAGATCA AATTTATGCA TATGAAACGT GATATCCACC
121 GTTATTCTCC CTCTCGAACA CAGGACTTCG ATAGTATCTT TTGTAATCTG TTCAGCTCAA
181 CTATTTGTTA CCATACCATC ATCATCACTT TACACATACT CATCATGTGT TTATTGACGC
241 GAGCGCGACT TGCAAGAAAA GGCATAATTG TGGCGAGGTA ATGAAAAATT TTTGACGTTT
301 CCCTCATTCA TTCATACATA ACATTTTGGG ATTTTGGAAC AAGGCGAGAG GAGAACGTCT
361 TTGGACCACT GTAATAGTAC ACAATGCAAG ATATTTTAAG TTTATGCTAA AAATCCAGAA
421 GTGACGCTTC ATCAACGGTG TCAATATGGA CCGAATTTTA GTGTATCTTC AATTGTAACC
481 GTGAGGAGTA GACTCTTTGA ATACGGGGGA AGAAGAAATA TCATATTCAA AGCTAATTCA
541 TTGAAATTAG TGCTTGTCTC ATCTAGCCTT TAGTGCTTAA TCTCTGGAGG AGCACATATG
601 GGGTTAAAGC CATGCCGGGA CTGGGGGCCC CTATCGGGGC TCGAACCCGA ATCCCGCGAG
661 TATTTATTTG AAGGTCCGGG ACGCAAGTTA CCTAATCTGG TTAATTGATA TCCCATTTAG
721 GCGATGACGT TCCTTCCCCT CACCCCTCGG CTTGTTAGAA GATCTATTGT TATAGCCTCC
781 TCTGGAAGAA TTTATGCCAG ATGAAGAAAA AAACTTCTCG AAGTTCCCAG ATGCCCAAAT
841 GAGGGCTTTC CATCCCTGTT AGCTGGAAAA GTGTAAGTAT ATCTATATAA AAAGTCGGCC
901 TACTTTTGCC AGGTTCGTCT TTCACTTGCA CTCTCTTGAT CTTACTTTCT ACTCAAAAAG
961 AATCCAATAC ACAAAAATAA AATCAGTACT ATTACTAATA
```

***TOS3* (1.000 bp)**

```
  1 CCACCCAGTT GGCAGCGGAT GCCGAAGGGC GGAAGGAAGG GGCGTGTATA GGCTTCTTTA
 61 CGAGAGCAAA AGACATTACA ACGTACAAGG GACATTCCGT TCCCTTGTAC GTCATGTTAA
121 TGAGCAAGAA ACCGCTCACC GGCAATGGTT GGTGGTAGTG GTAGTATGGA CTCTTTGGGT
181 GATGAAAAGT AATCAGAACA TTCTGCAATT TCTTTACATT TCCAATACTT GCTCCATTTA
241 ATCCTTTCCT TAGGTGGCGA TAGCCCACGC GAAAGACGAC CTAGGGTCAG GAGATTATCA
301 ATGCACTTGA GGCTTTAACT ACTATGGCTT AAGAAGGGTA GCTACTTTAT TTTTGTGCTT
361 TTAAGAAATG TAATCATTAA TTAAGTAATT AGGAGGTCAA GACGAAAACC ATAAATAGAT
421 TGTGTAGAAG GGGCCGTACA TTTTGTTTCT TATGCAATGT GGTAGTTTCG TTTACCGCCC
481 TCGCCGGTCC ATCACAGCAT TTTGGAGTTC CTTTCTAAGG TCCATCGGAG CACCTTTATT
541 TTTTTTGCGC TTCGGATAAA TTGCATTTGG TTGATAACTT CCATCTCCAC CCGTCCCCTC
601 GCACCGGACC TTTTAAAGAT CCCTTTGTAT ATAATAAACC AACGGGTCCT ATAAAGACTA
661 CCGCTGATAT CTTCGGTTTG CACAACGTCC TTCAGCAAGC AAAATATGGC TGTTTTTATT
721 CTCTGTATGT CCTCATTGAT GAGGACTCGC AACAATCTTG GCTATTTTTT TCGTTGAGCA
781 TACCCGGTCC CTGAATCTTT TGTGCGGGTA TTATACGAAG AGCGGGTGTG CCTTACTTAT
841 CCGCGCGGTA TATAAAGCCA TACATTGTTA GGACAGGTGT CCCCAATAAG AAACACTACT
901 TGTAAGTGGT ATATAATGCT CGAGCCTCTA GTGACTCGTA CTCTACGCCT TGAAATTGCT
961 GCGCACATAT TCTGCATATA AAAAGGAAGC TTTGAAGAAT
```

***TPO2* (1.000 bp)**

```
  1 CCTATGCAAA AACCCTTCCC CTGATCTATT TGCCATTTAG CCGCCCAAAG TACCGCAGAA
 61 GCTGTTACAA ACACATCACG GGCCACCGGA AAAAGTATTT TTTTGCTGCT TACCTGAATA
121 TATTTGCGCT TAAAAAAATT TTCCGGCGCT TCCGAGTGAA ATATACTGCT TAACTGTGTC
181 CGGAGAAACC TCGTGCGGAA AACAGCGCAA ACCGCAAAAA CCCCGCAATG GGCGCGCTTA
241 CTCAAATTCC CTCGGGTGGT CTCACGGGCT GGCCGCAGCG CAGTCGCCTG CCGCACTAAA
301 CCTTAAAAAT GCGAGAGCGC ATGCACGGCG GACGTCGTGG AAGCCTGACG CGTGCGCCCT
361 TCTCCTCCCG AGGGACATAA TGTCTTGGTG CCTTACTAGA ACCGATTTCT CGAGATGATT
421 CCATAGCCGT TAAATTCATC TCAAAAGAAT AGAGAACACA GCAGCGCAGA ACGAAAACAA
481 ACTGTCGCGT CGCATTTTAC TGAACGAGTC ATTCGCATAT TCTGCTTAGG TGTCTGAGTC
541 TTTTAAACGC ATTCTAGGTT TTTCGAAAAA GCCACCGAAG AACACTGCCA TCCGCATGCA
601 CTGTAATGCC ACTGTTTATT TTTTTTTCTT GCTATATATA TACATTGCGC ATACACCCTT
661 TTGAGGTTTC GTACTAGGAT TTTTTTGTAT TTGTTTATGT TCTTTTTTCC TGAAGACATC
721 AAGAAGAAAT AAGAAAACAA ATATATCATT TTATTGTTTG TGTTTATAGA GAACAATATA
781 CAACTTCCTT CTCCTACTAT ATCAAGTTTA ATATTTAATT TTTTTTTTTT TTCGGTTTGA
841 ATTTTAATTT TAACTTTAAT TTCAAAAAGT AATTGATACA TTCTTTTATC AAAGCCCTGC
901 TATTATTCTT TATTTCTACA CCCTAATATT TTTTGTCCAT TGATATTTTT CCTTTAATTC
961 CATCTAATAA CTAATCACAA AAATAATACA CAAAACAAAT
```

***TPO3* (805 bp)**

```
  1 AGCAACATAA TTGACTGACC CACAATTTGG TTAGAGACAT GCTATGCTGA TCCTGCATAT
 61 CTCAAACAGG CAATATGATA ACATACATAA GCAATCTCGG CTACGCGGAT TTTGTGTGGG
121 TCTGAAAAAA AAAAAAAAAA AAAAAAAAAA TAGCGCGGAG CCGGGTGAGA ACGTTGGACG
181 GGTAACATAA AAACAGAGCT CTTTATATAA TCAAGCATTC CCTCCATACA AAATGTGTCT
241 GCAGCCACGT GCAAAGTACG GTCGTCGTGT GCTAAAGACC AGTAAGTACA GTCTAGCGGG
301 CCTCTGACTA GCTTCTCTGT GCTTGGCGAG GGGTTTACTG GAGCCCAATC GGACTAGCCG
361 GGCTTCGTCA CTGCGGCAAT GTCTTCGCCC AACGGATCGG AACAGCTCAC TGATTTCACC
421 CAAACGGGAA AAAGGAAAAA CAAAAACAAA ACAGAACGGC GTAGTTTCCG GCAGCCATGA
481 TGATCCCTGA TCCATAGAGC CATTTTTTTC ATTTATTTCC TAATGCATTT TGTTCATATT
541 CTGGATCTGC GTTAAACGCT GTAAAACTGG CTCGTTTCTT TTATATATCT TGCAATCATT
601 CGCTGCTCAA CCATCCTAGT CTCTTTTTAC ACGTACCCTT TTTCCACTTT TTCTTTTAGT
661 TTATATATTC TTTTCGCCTA AGAACATTTG TCTTTGTAAT CTACTTAACT TAGCAATTGT
721 CAGCTGTCCT GTTACAACCT GTTCTCCAAA GTGAATACAA TAAGCAGTAT TTGCTCTTTT
781 TCGTCTAGGT GCGTTGATCT AATCTTCTTT TTATTTTTCT TCTTCGTTCA GCCTTTTTTG
841 TCCTCACCGG TAGCGTTCAC CCTAATTCTT TAATTGTCAT ACCCATAATT ATACTACAAA
901 TTTCGTGGTC TAGTTTTCTA ATATAACTAA TTACTTTCTC CCTTCTTACT TCATTATTTT
961 AATTTTGCAT TAGTACTCCT CTAGCCAAAG ATAAACAGAA
```

***YGP1* (1.000 bp)**

```
  1 AGCGTGCTAT TTTTTAAAAA GGGCAAATTT AGGAATGTCT GACGGAATCC CTAATCATCT
 61 TTGGCTTTTC CCTTCACTCC CTTCTTTTAC CGACGATGGT TTGAAGGAAA GAGAAAAATG
121 AGCAATAGGA AAATTCAGTA GTCGATTTCC ATTTCAATTC CCTATTGGGT AATGTGGCGG
181 CGAGGGCTAA ACATTCGGTA TCATTGATTC AATGTTCTCA CAATGTTCAA ATTCTCGGAT
241 CGGGTATCCC TAATTGGGAT CTCTTTCCAG AGGAAGGGGC CTTGATGTGG GATTGAAGAC
301 ACATGAGTTT TTTTCTCACC GTCGAAACTT TTGCTTACCC GTAAAACATC GGCCATTCGT
361 TGGTTTATTA TGTGACTTTT TTGTGACTTT TTTTTTTGCG AAGTTTTCTC GTCATCCGCG
421 GAAGATCCGG CGGGCGAAAA AGTCTCTCAC TTTTACCCAA TCGGGGCATG TTATGTGGAC
481 ATGTTATGTG TCCAAGAGAC ACATGAGCCC AAAAAGGGTA CACTGTCTAA ATAGTTTACC
541 CTTTACGAAT CGAAACTTCT CGAAGAAGGG GCCGAAAATT AGCGCAGAAG TTGACGAAAG
601 TTTATACTTT TCCTAATGGA GAGGCTCAGG AGCTGAAAAG GGATATATTC GTACTCTATT
661 GCATCTTCAA ACTCCGAAGA ATCTCAGTAG GGAGTGAAGC CGGCTTCTCG ATGCTACAGT
721 TCAACATCGA TACTTTGAAG AAAGAAAGCG CCTATTATAT CTCTTTTACC CTATTAGTAA
781 TAATTAGGAA AAAGGGAGAA AAAAGTACCT CACTAAAAAA ACCATCATCT CTGAAATATA
841 AAAAGCTTGA TAGAGGGTGA CATTTGCTAG AACTTCTGCT GTGTTCTCTT GGGTTATTGC
901 TCTTATTGAA TATCCCTTCT ATTTCTTTCT TGCTTGTAAA AAATCAGCTC AAAAAACATC
961 TACAGGATTA ATCGTCAGTT AAGTAATACA GTAATAGAAA
```

***YRO2* (1.000 bp)**

```
  1 AACTTAGCTA TGTAGGCCGG TGTGTTTGTT CATATCACGG CATACGTTAA CACAATGTAA
 61 ATCTTGCTTT GGGTTGACTG AGGGAAATAA CTATAGACAT CACACCAATG GCTAAGGGAT
121 ATTTCGACCT CGCTATGGGT TCTAAATGAG GGAATGGAAA AAAAGGGTAA ATTGAAAACA
181 ACCTACCGAT TTTGGTACCA CTTCCGTGAT TTTCGATTCT TCAATTTCCG CATAATTTGC
241 CTCAAATAGG AAATCTGAGC TGTTTTTTTC TTACCCTCGA GTGGTCGTTC TGCGGACTTT
301 TCTCCGGTCC AATGCATGAA TCCCTTTAAG AAGCATGAAG AAAAAAAAAA ATAGCCTCTT
361 GTAAAAGCAA AAATGCCTTT CACTATTTCT GTGTAAACGA ATAATTTCGT CATCTTAGTA
421 GTTCACTGGA TAATTGTGCT TTCCAATCAG ATTAGTTAGT AAAACCCCTA GAAAATTTCA
481 ATGTTAGATC GGGATGATTC CTCATCGGGT TGTTGCTTAT TCATGATCCT TTCAATTACT
541 GGAAGCTTAC CGAAAAGATA TATAAAGAAC CGAACTTATT CGGTTTACAT GTAAGACAGT
601 GTGTTAGTTA TGGTTTTCCG CCCTTTTCTT CTCTTGTTTT CTTCGATATT TGTTATTAAC
661 CTTTTCTCAT TTACGATTAT ATAATCAAGT GTCAAGATTG TGTCAGCAAA TAACAGTAAT
721 ATTCCAACCC GAAAAAAACT ATCTATTTAT CTTTGGATAC TTTAACATAG ACAAGAAGTC
781 CTAAGAACTT ACCATAATTT TTTTTCTAAA AACCCTTTTT TACTCAAAAC TAATAGCTTA
841 TTCTGGTTAT TTTTAATCAA TCTAGAATTT TTTTCTTTTA ATTCCAAACA ATCTAATAAC
901 GTTTAACCAC TAATTCTTTA ATTATTAATA TTTAATTTTA AAAATCTTCT AATTTTCCTG
961 ATAACTTAAA GTGACACTAT TTTTTAAAAA AAGCATCAAA
```

3. Intracellular pH value

We determined the influence of different acetic acid concentrations in the minimal medium on the cytoplasmatic pH value (pH_i) of yeast cells by using a pH-sensitive green fluorescent protein (pHluorin, [2]). The experimental setup was based on the publication of Orij *et al.* 2009 [3].

Briefly, *S.c.* BY4742 cells where transformed with the plasmid pYES-PACT1-pHluorin (kindly provided by Gertien Smits, University of Amsterdam). For each measurement an *in-situ* calibration with known pH value was carried out. *S.c.* BY4742 wild type strain was used to measure background fluorescence. Cells were cultured over night respectively for 24 h in media with or without containing acetic acid at different concentrations. OD_{600} was determined and adjusted to an optical density of 1. After centrifugation the cell pellet was resuspended in digitonin solution (100 µg/ ml PBS) and incubated for 10 min at room temperature for permeabilisation. Fluorescence was measured at an emission of 512 nm and two different extinction wavelengths of 390 nm and 470 nm in a black 96-well plate with a transparent bottom using TECAN infinite M200 pro. For the *in-situ* calibration citric acid phosphate buffer, for the samples ddH_2O was used.

After normalization of the measured fluorescence to a OD_{600} of 1, the fluorescence signal of the wild-type strain was subtracted and the ratio of the fluorescence signals at 390 nm and 470 nm (two extinction maxima of the fluorescent protein used) was determined. By plotting the pH value against the ratio of the fluorescence signals, the intracellular pH value of the sample could be determined.

Suppl. Table 1: Determination of the intracellular pH value (pH_i) of yeast cells using pH sensitive green fluorescence protein pHlourin incubating in minimal media pH 4 containig no respectively 5, 15 or 30 mM of acetic acid. The mean value of three independent measurements is shown.

**incubation time**	**acetic acid**	**determined pH_i**
24 h	0 mM	6,97
	5 mM	7,02
	15 mM	7,02
	30 mM	7,50
over night	0 mM	7,02
	5 mM	6,97
	15 mM	6,87
	30 mM	7,08

After 24 h of incubation in medium containing up to 30 mM of acetic acid, the intracellular pH value remained nearly stable in a neutral range. Therefore we concluded that the pH of the medium had no significant impact on the performance of the sensor system. These results were similar to those of other authors, *e.g.* Orij *et al.* 2009 and Brett *et al.* 2005 [3, 4].

4. References supplements

[1] Mira, N.P., Henriques, S.F., Keller, G., Teixeira, M.C., *et al*., Identification of a DNA-binding site for the transcription factor Haa1, required for *Saccharomyces cerevisiae* response to acetic acid stress. *Nucleic Acids Res.* 2011, 1-12.

[2] Miesenböck, G., De Angelis, D.A., Rothman, J.E., Visualizing secretion and synaptic transmission with pH-sensitive green fluorescent proteins. *Nature* 1998, *394*, 192-195.

[3] Orij, R., Postmus, J., Ter Beek, A., Brul, S., *et al*., In vivo measurement of cytosolic and mitochondrial pH using a pH-sensitive GFP derivative in *Saccharomyces cerevisiae* reveals a relation between intracellular pH and growth. *Microbiol. Read. Engl.* 2009, *155*, 268-278.

[4] Brett, C.L., Tukaye, D.N., Mukherjee, S., Rao, R., The yeast endosomal $Na^{+}K^{+}/H^{+}$ exchanger Nhx1 regulates cellular pH to control vesicle trafficking. *Mol. Biol. Cell* 2005, *16*, 1396-1405.

Row Data Figure 2

				YGP1			YRO2			TPO2			SPI1			TPO3			PHM8			TOS3		
			t in s	43462	43496	43532	43462	43496	43532	43462	43496	43532	43462	43496	43532	43457	43507	43534	43457	43507	43534	43457	43507	43534
			t in min	724	725	726	724	725	726	724	725	726	724	725	726	724	725	726	724	725	726	724	725	726
			t in h	12	12	12	12	12	12	12	12	12	12	12	12	12	12	12	12	12	12	12	12	12
S.c. BY4742 + p426-X-tRFP	C01	XA2	1	298	230	237	307	212	215	459	393	437	126	128	no data	1356	1197	1112	475	291	386	1290	1389	1332
0 mM acetic acid	C01	XA2	2	1,6234	1,7025	1,8564	1,5218	1,6949	1,9211	1,6931	1,7673	1,8698	1,634	1,7156	no data	1,7933	1,7098	1,6986	1,7306	1,7349	1,7912	1,7533	1,704	1,8006
	C02	XA2	1	248	232	263	295	228	208	472	427	380	123	108	125	1309	1158	1105	453	297	360	1324	1337	1285
	C02	XA2	2	1,5187	1,7203	1,849	1,487	1,6799	1,8197	1,6999	1,7842	1,8267	1,5954	1,6726	1,915	1,7588	1,7014	1,6766	1,6832	1,6993	1,7379	1,7611	1,6995	1,7865
	C03	XA2	1	245	211	245	293	224	200	465	402	408	141	108	122	1353	1100	1071	445	284	362	1346	1345	1336
	C03	XA2	2	1,4695	1,6175	1,7693	1,505	1,6466	1,7948	1,6131	1,7131	1,7906	1,6203	1,6767	1,8208	1,7209	1,6676	1,6575	1,6479	1,6469	1,711	1,7167	1,6743	1,7407
			mean FL	264	224	248	298	221	208	465	407	408	130	115	124	1339	1152	1096	458	291	369	1320	1357	1318
			mean Abs	1,537	1,680	1,825	1,505	1,674	1,845	1,669	1,755	1,829	1,617	1,688	1,868	1,758	1,693	1,678	1,687	1,694	1,747	1,744	1,693	1,776
Relative Fluorescence	0 mM AA			172	134	136	198	132	113	279	232	223	80	68	66	762	680	653	271	172	211	757	802	742
S.c. BY4742 + p426-X-tRFP	C04	XB2	1	907	768	992	617	595	715	1785	1423	1433	170	152	164	1490	1108	1089	678	454	510	1439	1419	1286
5 mM acetic acid	C04	XB2	2	1,4846	1,5199	1,7302	1,3568	1,6666	1,8112	1,5582	1,746	1,7998	1,46	1,67	1,8165	1,7739	1,6233	1,5934	1,6396	1,6613	1,6477	1,7439	1,6532	1,643
	C05	XB2	1	869	842	946	593	567	751	1599	1427	1463	163	147	157	1502	1165	1154	665	419	493	1449	1376	1325
	C05	XB2	2	1,4687	1,5674	1,7243	1,3743	1,5468	1,7909	1,5313	1,7064	1,7927	1,4698	1,6558	1,829	1,7201	1,604	1,611	1,638	1,6465	1,6696	1,703	1,6354	1,6945
	C06	XB2	1	822	799	879	615	519	692	1685	1245	1418	159	149	152	1492	1169	1115	685	388	515	1404	1326	1390
	C06	XB2	2	1,4021	1,5042	1,6938	1,371	1,5335	1,7547	1,4951	1,6344	1,7311	1,4696	1,6047	1,8264	1,7098	1,5487	1,5556	1,5443	1,6175	1,6271	1,574	1,5916	1,6799
			mean FL	866	803	939	608	560	719	1690	1365	1438	164	149	158	1495	1147	1119	676	420	506	1431	1374	1334
			mean Abs	1,452	1,531	1,716	1,367	1,582	1,786	1,528	1,696	1,775	1,466	1,644	1,824	1,735	1,592	1,587	1,607	1,642	1,648	1,674	1,627	1,672
Relative Fluorescence	5 mM AA			597	525	547	445	354	403	1106	805	810	112	91	86	862	721	705	421	256	307	855	844	797
S.c. BY4742 + p426-X-tRFP	C07	XC2	1	2025	1713	2335	1261	1095	1587	2835	2187	2379	199	182	226	1456	1107	1054	929	602	737	1558	1325	1583
15 mM acetic acid	C07	XC2	2	1,2755	1,3129	1,5586	1,2141	1,388	1,6189	1,3539	1,4388	1,6087	1,346	1,4119	1,7021	1,6156	1,4459	1,4793	1,4686	1,4978	1,4874	1,5522	1,4737	1,5517
	C08	XC2	1	1998	1871	2345	1274	1054	1603	2685	2387	2308	230	178	219	1559	1142	1100	910	589	726	1493	1257	1389
	C08	XC2	2	1,2498	1,3735	1,5711	1,2724	1,3909	1,6324	1,3335	1,5253	1,6467	1,331	1,4248	1,6786	1,5922	1,4952	1,4074	1,4831	1,5314	1,4901	1,6034	1,4916	1,5084
	C09	XC2	1	2019	1811	2357	1160	1091	1611	2770	2484	2326	198	166	200	1488	1159	1102	874	596	646	1532	1441	1485
	C09	XC2	2	1,3226	1,4103	1,6445	1,2548	1,3767	1,6413	1,3822	1,5226	1,629	1,3965	1,4774	1,7481	1,6408	1,4595	1,4252	1,4964	1,5514	1,4887	1,5797	1,5371	1,5853
			mean FL	2014	1798	2346	1232	1080	1600	2763	2353	2338	209	175	215	1501	1136	1085	904	596	703	1528	1341	1486
			mean Abs	1,283	1,366	1,591	1,247	1,385	1,631	1,357	1,496	1,628	1,358	1,438	1,710	1,616	1,467	1,437	1,483	1,527	1,489	1,578	1,501	1,548
Relative Fluorescence	15 mM AA			1570	1317	1474	988	780	981	2037	1573	1436	154	122	126	929	774	755	610	390	472	968	894	959
S.c. BY4742 + p426-X-tRFP	C10	XD2	1	2618	2341	2897	1228	1116	1802	2370	1994	2029	268	206	324	1099	862	807	965	728	815	1374	1270	1439
30 mM acetic acid	C10	XD2	2	1,0608	1,2302	1,3301	1,0431	1,1851	1,3773	1,1817	1,3155	1,33	1,2179	1,1851	1,5044	1,4025	1,2126	1,2084	1,3178	1,336	1,3221	1,3852	1,2552	1,3973
	C11	XD2	1	2728	2318	3074	1198	1115	1823	2327	1760	1890	245	218	299	1014	847	810	1038	709	744	1300	1280	1449
	C11	XD2	2	1,1009	1,2174	1,3488	1,0328	1,1817	1,3832	1,2162	1,2632	1,3698	1,1664	1,2387	1,4836	1,3635	1,2222	1,222	1,3286	1,3283	1,2609	1,3348	1,2293	1,3138
	C12	XD2	1	2616	2150	3153	1299	1083	1655	2313	1832	1920	248	186	294	1039	823	758	922	670	756	1212	1237	1364
	C12	XD2	2	1,0904	1,1897	1,3804	1,12	1,1695	1,4334	1,211	1,2899	1,3628	1,2031	1,2397	1,4833	1,3598	1,2481	1,2244	1,3373	1,3356	1,3353	1,3627	1,2816	1,3756
			mean FL	2654	2270	3041	1242	1105	1760	2337	1862	1946	254	203	306	1051	844	792	975	702	772	1295	1262	1417
			mean Abs	1,084	1,212	1,353	1,065	1,179	1,398	1,203	1,290	1,354	1,196	1,221	1,490	1,375	1,228	1,218	1,328	1,333	1,306	1,361	1,255	1,362
Relative Fluorescence	30 mM AA			2448	1872	2248	1166	937	1259	1942	1444	1437	212	167	205	764	688	650	734	527	591	952	1006	1040
x-fold induction	MM4 5/ MM4			3,48	3,93	4,02	2,24	2,68	3,58	3,96	3,47	3,63	1,39	1,34	1,31	1,13	1,06	1,08	1,55	1,49	1,45	1,13	1,05	1,07
	MM4 15/ MM4			9,15	9,86	10,83	4,98	5,90	8,72	7,30	6,78	6,43	1,91	1,80	1,90	1,22	1,14	1,16	2,25	2,27	2,23	1,28	1,11	1,29
	MM4 30/ MM4			14,27	14,02	16,52	5,88	7,09	11,19	6,97	6,22	6,44	2,64	2,45	3,10	1,00	1,01	0,99	2,71	3,07	2,79	1,26	1,25	1,40

FL: Fluorescence
Abs: Absorbance
MM: Minimal Medium
AA: Acetic Acid

Row Data Figure 3

			t in s	0	7240	14485	21727	28972	36215	43462	50706	57952	65199	72446	79694
Measurement 1			t in min	0	121	241	362	483	604	724	845	966	1087	1207	1328
			t in h	0	2	4	6	8	10	12	14	16	18	20	22
S.c. BY4742 + p426-YGP1-tRFP	C01	XA2	1	289	267	267	248	255	227	298	321	350	377	440	531
**0 mM acetic acid**	C01	XA2	2	0,6054	0,6707	0,7356	0,8457	1,1099	1,3214	1,6234	1,7969	1,9715	2,0261	2,0477	2,0799
	C02	XA2	1	272	249	244	252	231	244	248	297	310	365	389	461
	C02	XA2	2	0,6049	0,6579	0,7151	0,8139	0,9938	1,2153	1,5187	1,7491	1,8938	2,006	2,0345	2,0571
	C03	XA2	1	281	272	260	249	248	229	245	287	319	364	377	458
	C03	XA2	2	0,594	0,6516	0,694	0,8137	0,96	1,2148	1,4695	1,7207	1,8779	1,9975	2,0247	2,0571
			mean FL	281	263	257	250	245	233	264	302	326	369	402	483
			mean Abs	0,601	0,660	0,715	0,824	1,021	1,251	1,537	1,756	1,914	2,010	2,036	2,065
Relative Fluorescence	0 mM AA			467	398	359	303	240	187	172	172	170	183	197	234
BY4742 + YGP1	C04	XB2	1	290	291	324	414	493	658	907	1194	1297	1345	1337	1423
**5 mM acetic acid**	C04	XB2	2	0,6196	0,6449	0,7015	0,7812	1,0176	1,2292	1,4846	1,7298	1,8763	1,9751	2,0397	2,0669
	C05	XB2	1	297	310	322	377	459	609	869	1083	1223	1276	1287	1367
	C05	XB2	2	0,5988	0,6345	0,6856	0,7731	1,0046	1,2121	1,4687	1,7194	1,8843	1,9607	2,0359	2,0816
	C06	XB2	1	308	277	348	386	473	592	822	1013	1186	1249	1263	1289
	C06	XB2	2	0,6164	0,6791	0,7145	0,8088	0,9272	1,1922	1,4021	1,6636	1,8395	1,9186	1,995	2,0412
			mean FL	298	293	331	392	475	620	866	1097	1235	1290	1296	1360
			mean Abs	0,612	0,653	0,701	0,788	0,983	1,211	1,452	1,704	1,867	1,951	2,024	2,063
Relative Fluorescence	5 mM AA			488	448	473	498	483	512	597	643	662	661	640	659
S.c. BY4742 + p426-YGP1-tRFP	C07	XC2	1	322	341	465	659	974	1406	2025	2797	3376	3693	3717	3786
**15 mM acetic acid**	C07	XC2	2	0,6512	0,6631	0,6992	0,753	0,9052	1,0684	1,2755	1,4793	1,735	1,8593	1,9424	1,9887
	C08	XC2	1	312	309	442	658	959	1337	1998	2798	3229	3741	3975	4146
	C08	XC2	2	0,6047	0,6926	0,7272	0,7899	0,902	1,0641	1,2498	1,4957	1,7371	1,8779	1,9355	2,0237
	C09	XC2	1	326	327	446	658	975	1422	2019	2824	3257	3655	3812	3955
	C09	XC2	2	0,5919	0,6663	0,7328	0,7946	0,8879	1,072	1,3226	1,5709	1,7518	1,8855	1,9391	2,024
			mean FL	320	326	451	658	969	1388	2014	2806	3287	3696	3835	3962
			mean Abs	0,616	0,674	0,720	0,779	0,898	1,068	1,283	1,515	1,741	1,874	1,939	2,012
Relative Fluorescence	15 mM AA			520	483	627	845	1079	1300	1570	1852	1888	1972	1978	1969
S.c. BY4742 + p426-YGP1-tRFP	C10	XD2	1	305	314	490	762	1288	1872	2618	3692	4793	5868	6973	7546
**30 mM acetic acid**	C10	XD2	2	0,4827	0,5051	0,5361	0,7375	0,8464	0,8746	1,0608	1,2299	1,4354	1,6594	1,8287	1,9414
	C11	XD2	1	305	322	498	817	1375	2014	2728	3825	5013	6196	7408	7936
	C11	XD2	2	0,5194	0,5231	0,5614	0,6924	0,7777	0,8483	1,1009	1,2192	1,4165	1,6578	1,8304	1,9376
	C12	XD2	1	297	317	458	796	1249	1882	2616	3690	4962	6140	7124	7549
	C12	XD2	2	0,5565	0,6021	0,6682	0,7698	0,7926	0,8711	1,0904	1,2633	1,4526	1,6608	1,8116	1,9178
			mean FL	302	318	482	792	1304	1923	2654	3736	4923	6068	7168	7677
			mean Abs	0,520	0,543	0,589	0,733	0,806	0,865	1,084	1,237	1,435	1,659	1,824	1,932
Relative Fluorescence	30 mM AA			582	585	819	1080	1619	2224	2448	3019	3431	3657	3931	3973
x-fold Induction	MM4 5/ MM4			1,05	1,13	1,32	1,64	2,02	2,74	3,48	3,74	3,88	3,60	3,24	2,82
	MM4 15/ MM4			1,11	1,21	1,74	2,79	4,50	6,97	9,15	10,78	11,07	10,75	10,01	8,41
	MM4 30/ MM4			1,25	1,47	2,28	3,57	6,76	11,92	14,27	17,57	20,13	19,94	19,91	16,97

			t in s	0	7249	14497	21746	28996	36247	43496	50746	57997	65248	72500	79753
Measurement 2			t in min	0	121	242	362	483	604	725	846	967	1087	1208	1329
			t in h	0	2	4	6	8	10	12	14	16	18	20	22
S.c. BY4742 + p426-YGP1-tRFP	C01	XA2	1	209	175	194	188	190	190	230	276	304	309	357	391
**0 mM acetic acid**	C01	XA2	2	0,7378	0,8048	0,8688	0,9874	1,2126	1,4677	1,7025	1,9052	2,0476	2,1124	2,1553	2,179
	C02	XA2	1	228	206	200	200	189	182	232	270	273	334	376	421
	C02	XA2	2	0,7783	0,8629	0,9128	1,0095	1,2051	1,4996	1,7203	1,9233	2,0551	2,0923	2,1179	2,1551
	C03	XA2	1	219	213	186	198	214	187	211	261	288	315	374	441
	C03	XA2	2	0,6161	0,7368	0,8137	0,9417	1,1246	1,3676	1,6175	1,8369	1,9765	2,0269	2,0578	2,1032
			mean FL	219	198	193	195	198	186	224	269	288	319	369	418
			mean Abs	0,711	0,802	0,865	0,980	1,181	1,445	1,680	1,888	2,026	2,077	2,110	2,146
Relative Fluorescence	0 mM AA			308	247	223	199	167	129	134	142	142	154	175	195
S.c. BY4742 + p426-YGP1-tRFP	C04	XB2	1	239	230	244	305	406	563	768	1024	1167	1349	1305	1349
**5 mM acetic acid**	C04	XB2	2	0,5018	0,5451	0,6103	0,7112	0,9926	1,2656	1,5199	1,7719	1,9296	2,0452	2,0775	2,1194
	C05	XB2	1	253	237	275	346	423	592	842	1047	1176	1210	1259	1297
	C05	XB2	2	0,6548	0,6701	0,7073	0,8947	1,0709	1,2955	1,5674	1,7967	1,9304	2,0243	2,0927	2,1448
	C06	XB2	1	240	237	253	305	416	594	799	1005	1105	1144	1240	1183
	C06	XB2	2	0,5504	0,5901	0,6875	0,838	1,0194	1,2759	1,5042	1,7875	1,9335	2,0108	2,0787	2,1267
			mean FL	244	235	257	319	415	583	803	1025	1149	1234	1268	1276
			mean Abs	0,569	0,602	0,668	0,815	1,028	1,279	1,531	1,785	1,931	2,027	2,083	2,130
Relative Fluorescence	5 mM AA			429	390	385	391	404	456	525	574	595	609	609	599
S.c. BY4742 + p426-YGP1-tRFP	C07	XC2	1	249	251	338	539	770	1161	1713	2438	2863	3224	3337	3336
**15 mM acetic acid**	C07	XC2	2	0,5185	0,6051	0,6968	0,8318	0,9344	1,1135	1,3129	1,5617	1,7912	1,9256	1,9983	2,0507
	C08	XC2	1	253	264	336	547	813	1242	1871	2554	2988	3336	3417	3471
	C08	XC2	2	0,6859	0,7302	0,7689	0,8964	0,9918	1,1963	1,3735	1,6377	1,8218	1,9366	2,0121	2,1004
	C09	XC2	1	245	258	348	548	831	1242	1811	2491	2805	3092	3133	3165
	C09	XC2	2	0,6513	0,7395	0,7447	0,8161	1,0579	1,2178	1,4103	1,6805	1,8143	1,9512	2,0499	2,0926
			mean FL	249	258	341	545	805	1215	1798	2494	2885	3217	3296	3324
			mean Abs	0,619	0,692	0,737	0,848	0,995	1,176	1,366	1,627	1,809	1,938	2,020	2,081
Relative Fluorescence	15 mM AA			403	373	462	642	809	1033	1317	1533	1595	1660	1631	1597
S.c. BY4742 + p426-YGP1-tRFP	C10	XD2	1	248	264	382	619	1071	1622	2341	3368	4342	5443	6290	6571
**30 mM acetic acid**	C10	XD2	2	0,5871	0,6046	0,6648	0,7757	0,7583	0,9365	1,2302	1,4022	1,6145	1,8089	1,9577	2,0455
	C11	XD2	1	259	261	357	633	1083	1665	2318	3279	4374	5347	6499	6874
	C11	XD2	2	0,5863	0,6132	0,6208	0,67	0,7714	0,9568	1,2174	1,3996	1,5881	1,779	1,9401	2,0595
	C12	XD2	1	244	259	346	574	939	1509	2150	3038	4181	5178	6082	6310
	C12	XD2	2	0,6271	0,6341	0,6746	0,7446	0,8257	0,9465	1,1897	1,381	1,5728	1,754	1,8897	2,0139
			mean FL	250	261	362	609	1031	1599	2270	3228	4299	5323	6290	6585
			mean Abs	0,600	0,617	0,653	0,730	0,785	0,947	1,212	1,394	1,592	1,781	1,929	2,040
Relative Fluorescence	30 mM AA			417	423	554	834	1313	1689	1872	2315	2701	2989	3261	3229
x-fold Induction	MM4 5/ MM4			1,39	1,58	1,72	1,96	2,41	3,53	3,93	4,03	4,18	3,96	3,48	3,08
	MM4 15/ MM4			1,31	1,51	2,07	3,22	4,83	8,01	9,86	10,77	11,21	10,80	9,33	8,21
	MM4 30/ MM4			1,36	1,71	2,48	4,18	7,84	13,10	14,02	16,26	18,98	19,44	18,65	16,59

			t in s	0	7256	14510	21765	29021	36276	43532	50776	58019	65262	72506	79751
Measurememt 3			t in min	0	121	242	363	484	605	726	846	967	1088	1208	1329
			t in h	0	2	4	6	8	10	12	14	16	18	20	22
S.c. BY4742 + p426-YGP1-tRFP	C01	XA2	1	172	147	157	155	153	174	237	270	304	381	408	434
**0 mM acetic acid**	C01	XA2	2	0,7952	0,9417	1,0295	1,1781	1,4164	1,6039	1,8564	2,0056	2,0693	2,1163	2,1588	2,1765
	C02	XA2	1	221	213	202	198	184	214	263	287	324	373	414	509
	C02	XA2	2	0,5472	0,6006	0,7461	1,0582	1,288	1,5838	1,849	1,9398	2,0548	2,1158	2,1438	2,1655
	C03	XA2	1	219	206	214	205	187	206	245	282	333	370	459	551
	C03	XA2	2	0,4573	0,4979	0,7006	0,9565	1,2338	1,5057	1,7693	1,8968	2,0035	2,061	2,0956	2,1086
			mean FL	204	189	191	186	175	198	248	280	320	375	427	498
			mean Abs	0,600	0,680	0,825	1,064	1,313	1,564	1,825	1,947	2,043	2,098	2,133	2,150
Relative Fluorescence				340	277	231	175	133	127	136	144	157	179	200	232
S.c. BY4742 + p426-YGP1-tRFP	C04	XB2	1	223	234	294	373	485	726	992	1156	1253	1283	1330	1340
**5 mM acetic acid**	C04	XB2	2	0,4745	0,4872	0,5712	0,8946	1,2122	1,4341	1,7302	1,896	1,9873	2,0772	2,1211	2,136
	C05	XB2	1	237	269	336	390	470	661	946	1085	1204	1236	1330	1310
	C05	XB2	2	0,5442	0,603	0,6432	0,9105	1,1717	1,4089	1,7243	1,8623	1,9568	2,0353	2,1274	2,1541
	C06	XB2	1	214	224	282	389	454	680	879	1033	1108	1160	1222	1260
	C06	XB2	2	0,4785	0,5203	0,5873	0,8975	1,1944	1,4005	1,6938	1,8861	1,9846	2,0569	2,1219	2,1531
			mean FL	225	242	304	384	470	689	939	1091	1188	1226	1294	1303
			mean Abs	0,499	0,537	0,601	0,901	1,193	1,415	1,716	1,881	1,976	2,056	2,123	2,148
Relative Fluorescence				450	451	506	426	394	487	547	580	601	596	609	607
S.c. BY4742 + p426-YGP1-tRFP	C07	XC2	1	224	278	471	740	1080	1630	2335	3030	3433	3572	3613	3593
**15 mM acetic acid**	C07	XC2	2	0,5843	0,7229	0,831	0,9365	1,1095	1,3207	1,5586	1,8055	1,9331	2,0262	2,1007	2,1106
	C08	XC2	1	230	255	423	691	1097	1643	2345	2990	3303	3496	3577	3571
	C08	XC2	2	0,6617	0,7511	0,8038	0,8845	1,1227	1,3269	1,5711	1,8193	1,9483	2,0297	2,0966	2,1557
	C09	XC2	1	203	253	372	699	1072	1593	2357	2997	3512	3655	3857	4053
	C09	XC2	2	0,7165	0,7267	0,7999	0,9863	1,1514	1,37	1,6445	1,8042	1,9307	2,0247	2,1091	2,1131
			mean FL	219	262	422	710	1083	1622	2346	3006	3416	3574	3682	3739
			mean Abs	0,654	0,734	0,812	0,936	1,128	1,339	1,591	1,810	1,937	2,027	2,102	2,126
Relative Fluorescence				335	357	520	759	960	1211	1474	1661	1763	1763	1752	1758
S.c. BY4742 + p426-YGP1-tRFP	C10	XD2	1	221	251	465	847	1384	1977	2897	3880	4991	6032	6451	6649
**30 mM acetic acid**	C10	XD2	2	0,5403	0,4971	0,5673	0,6436	0,8296	1,1159	1,3301	1,5486	1,7378	1,9202	2,0345	2,0642
	C11	XD2	1	235	259	485	866	1552	2157	3074	4283	5332	6124	6701	6594
	C11	XD2	2	0,5225	0,4694	0,6631	0,8078	0,8786	1,1721	1,3488	1,5501	1,7398	1,9028	2,0229	2,046
	C12	XD2	1	233	249	460	842	1367	2047	3153	4478	5762	7297	7890	8099
	C12	XD2	2	0,5928	0,5776	0,6727	0,8125	0,8649	1,1988	1,3804	1,589	1,7764	1,9676	2,0968	2,1279
			mean FL	230	253	470	852	1434	2060	3041	4214	5362	6484	7014	7114
			mean Abs	0,552	0,515	0,634	0,755	0,858	1,162	1,353	1,563	1,751	1,930	2,051	2,079
Relative Fluorescence	30 mM AA			416	492	741	1129	1672	1773	2248	2697	3061	3359	3419	3421
x-fold Induction	MM4 5/ MM4			1,32	1,63	2,19	2,44	2,96	3,85	4,02	4,04	3,83	3,34	3,04	2,62
	MM4 15/ MM4			0,98	1,29	2,25	4,34	7,22	9,57	10,83	11,57	11,24	9,87	8,75	7,59
	MM4 30/ MM4			1,22	1,77	3,20	6,46	12,57	14,01	16,52	18,78	19,52	18,81	17,08	14,77

FL: Fluorescence
Abs: Absorbance
MM: Minimal Medium
AA: Acetic Acid

Row Data Figure 4

measurement 1		t in h	0	4	8	12	16	20
S.c. BY4742 + p426-YGP1-tRFP	A02 XA2	1	264	231	219	325	372	439
0 mM acetic acid	A02 XA2	2	0.6294	0.8127	1.0818	1.6045	1.958	2.0474
	B02 XA14	1	261	222	216	285	354	365
	B02 XA14	2	0.5908	0.7364	1.1692	1.6672	1.9732	2.0829
		Average Fl	262.5	226.5	217.5	305	363	402
		Average Abs	0.6101	0.77455	1.1255	1.63585	1.9656	2.06515
Relative Fluorescence	0 mM AA		430	292	193	186	185	195
S.c. BY4742 + p426-YGP1-tRFP	E02 XA50	1	248	337	833	1826	2909	3311
15 mM acetic acid	E02 XA50	2	0.5927	0.6927	0.9505	1.45	1.8582	2.0333
	F02 XA62	1	246	341	806	1775	2911	3281
	F02 XA62	2	0.6663	0.7333	1.1216	1.4527	1.871	2.0529
		Average Fl	247	339	819.5	1800.5	2910	3296
		Average Abs	0.6295	0.713	1.03605	1.45135	1.8646	2.0431
Relative Fluorescence	15 mM AA		392	475	791	1241	1561	1613
x-fold Induction	15 mM		0,9	1,6	4,1	6,7	8,5	8,3

measurement 2		t in h	0	4	8	12	16	20
S.c. BY4742 + p426-YGP1-tRFP	A02 XA2	1	262	236	265	335	368	435
0 mM acetic acid	A02 XA2	2	0.6692	0.852	1.1514	1.7498	2.0164	2.0746
	B02 XA14	1	292	238	224	317	368	417
	B02 XA14	2	0.5979	0.7685	1.1658	1.7274	1.958	2.0515
		Average Fl	277	237	244.5	326	368	426
		Average Abs	0.63355	0.81025	1.1586	1.7386	1.9872	2.06305
Relative Fluorescence	0 mM AA		437	293	211	188	185	206
S.c. BY4742 + p426-YGP1-tRFP	E02 XA50	1	244	397	976	2118	3026	3257
15 mM acetic acid	E02 XA50	2	0.5453	0.706	1.1065	1.5295	1.915	2.0876
	F02 XA62	1	240	367	809	1879	2813	3028
	F02 XA62	2	0.5637	0.7487	1.3163	1.5277	1.8897	2.0474
		Average Fl	242	382	892.5	1998.5	2919.5	3142.5
		Average Abs	0.5545	0.72735	1.2114	1.5286	1.90235	2.0675
Relative Fluorescence	15 mM AA		436	525	737	1307	1535	1520
x-fold Induction	15 mM		1,0	1,8	3,5	7,0	8,3	7,4

measurement 3		t in h	0	4	8	12	16	20
S.c. BY4742 + p426-YGP1-tRFP	A02 XA2	1	258	205	185	260	410	795
0 mM acetic acid	A02 XA2	2	0.7045	0.9493	1.2123	1.7694	2.0833	2.1547
	B02 XA14	1	239	176	187	240	406	842
	B02 XA14	2	0.6946	0.9184	1.2978	1.8732	2.1264	2.1833
		Average Fl	248.5	190.5	186	250	408	818.5
		Average Abs	0.69955	0.93385	1.25505	1.8213	2.10485	2.169
Relative Fluorescence	0 mM AA		355	204	148	137	194	377
S.c. BY4742 + p426-YGP1-tRFP	E02 XA50	1	205	330	768	1609	2401	3972
15 mM acetic acid	E02 XA50	2	0.5709	0.7434	1.104	1.6063	1.9159	2.0618
	F02 XA62	1	197	321	708	1522	2008	2678
	F02 XA62	2	0.722	0.8869	1.1849	1.6279	1.996	2.1248
		Average Fl	201	325.5	738	1565.5	2204.5	3325
		Average Abs	0.64645	0.81515	1.14445	1.6171	1.95595	2.0933
Relative Fluorescence	15 mM AA		311	399	645	968	1127	1588
x-fold Induction	15 mM		0,9	2,0	4,4	7,1	5,8	4,2

measurement 1		t in h	0	4	8	12	16	20
absorbance	0 mM	A1	0,089	0,101	0,163	0,336	0,465	0,514
propionic acid	0 mM	A2	0,095	0,103	0,150	0,291	0,402	0,449
		Average	0,092	0,102	0,156	0,314	0,434	0,481
	2 mM	A7	0,086	0,109	0,172	0,369	0,506	0,493
	2 mM	A8	0,098	0,128	0,196	0,443	0,646	0,707
		Average	0,092	0,118	0,184	0,406	0,576	0,600
fluorescence	0 mM	Mean	991	317	240	245	253	283
propionic acid	0 mM	Mean	976	310	236	242	247	259
		Average	983,5	313,5	238	243,5	250	271
Relative Fluorescence	0 mM		10702	3086	1521	776	576	563
	2 mM	Mean	990	293	239	259	291	345
	2 mM	Mean	952	294	235	252	307	364
		Average	971	293,5	237	255,5	299	354,5
Relative Fluorescence	2 mM		10549	2483	1288	629	520	591
x-fold induction	2 mM		1,0	0,8	0,8	0,8	0,9	1,0

measurement 2		t in h	0	4	8	12	16	20
absorbance	0 mM	A1	0,086	0,091	0,144	0,274	0,398	0,446
propionic acid	0 mM	A2	0,089	0,096	0,147	0,274	0,386	0,434
		Average	0,088	0,093	0,145	0,274	0,392	0,440
	2 mM	A7	0,094	0,103	0,152	0,329	0,452	0,541
	2 mM	A8	0,094	0,102	0,155	0,315	0,514	0,537
		Average	0,094	0,102	0,154	0,322	0,483	0,539
fluorescence	0 mM	Mean	378	345	284	306	207	204
propionic acid	0 mM	Mean	392	350	281	307	202	218
		Average	385	347,5	282,5	306,5	204,5	211
Relative Fluorescence	0 mM		4385	3723	1945	1119	522	479
	2 mM	Mean	373	341	289	328	250	322
	2 mM	Mean	373	321	275	315	257	305
		Average	373	331	282	321,5	253,5	313,5
Relative Fluorescence	2 mM		3979	3237	1834	998	525	582
x-fold induction	2 mM		0,9	0,9	0,9	0,9	1,0	1,2

measurement 3		t in h	0	4	8	12	16	20
absorbance	0 mM	A1	0,089	0,091	0,136	0,274	0,419	0,476
propionic acid	0 mM	A2	0,099	0,101	0,147	0,292	0,428	0,481
		Average	0,094	0,096	0,142	0,283	0,423	0,479
	2 mM	A7	0,094	0,105	0,149	0,319	0,428	0,444
	2 mM	A8	0,091	0,101	0,149	0,294	0,434	0,486
		Average	0,092	0,103	0,149	0,306	0,431	0,465
fluorescence	0 mM	Mean	443	373	305	312	337	343
propionic acid	0 mM	Mean	420	377	304	321	338	351
		Average	431,5	375	304,5	316,5	337,5	347
Relative Fluorescence	0 mM		4598	3906	2150	1119	797	725
	2 mM	Mean	426	365	314	348	385	403
	2 mM	Mean	425	358	295	329	365	388
		Average	425,5	361,5	304,5	338,5	375	395,5
Relative Fluorescence	2 mM		4615	3518	2043	1105	869	851
x-fold induction	2 mM		1,0	0,9	1,0	1,0	1,1	1,2

measurement 1	mM	t in h	0	4	8	12	16	20
absorbance	0	B1	0,089	0,105	0,179	0,354	0,515	0,577
butyric acid	0	B2	0,086	0,105	0,168	0,338	0,470	0,524
		Average	0,088	0,105	0,173	0,346	0,492	0,551
	1	B5	0,086	0,105	0,163	0,341	0,519	0,594
	1	B6	0,089	0,102	0,164	0,322	0,477	0,474
		Average	0,087	0,104	0,164	0,331	0,498	0,534
fluorescence	0	Mean	908	307	241	247	249	269
butyric acid	0	Mean	946	297	236	239	239	248
		Average	927	302	238,5	243	244	258,5
Relative Fluorescence	0		10552	2878	1377	702	496	469
	1	Mean	964	315	240	251	286	355
	1	Mean	1004	313	260	249	264	299
		Average	984	314	250	250	275	327
Relative Fluorescence	1		11304	3025	1525	754	552	612
x-fold induction	1 mM		1,1	1,1	1,1	1,1	1,1	1,3

measurement 2	mM	t in h	0	4	8	12	16	20
absorbance	0	B1	0,089	0,102	0,155	0,283	0,395	0,442
butyric acid	0	B2	0,087	0,099	0,164	0,307	0,424	0,474
		Average	0,088	0,100	0,160	0,295	0,409	0,458
	1	B5	0,087	0,097	0,155	0,303	0,457	0,603
	1	B6	0,090	0,094	0,152	0,282	0,462	0,590
		Average	0,089	0,096	0,154	0,292	0,460	0,596
fluorescence	0	Mean	373	337	279	302	201	218
butyric acid	0	Mean	371	340	286	307	204	211
		Average	372	338,5	282,5	304,5	202,5	214,5
Relative Fluorescence	0		4215	3378	1770	1031	495	468
	1	Mean	379	342	289	307	206	219
	1	Mean	389	337	311	309	229	282
		Average	384	339,5	300	308	217,5	250,5
Relative Fluorescence	1		4322	3549	1954	1054	473	420
x-fold induction	1 mM		1,0	1,1	1,1	1,0	1,0	0,9

measurement 3	mM	t in h	0	4	8	12	16	20
absorbance	0	B1	0,086	0,097	0,146	0,279	0,407	0,455
butyric acid	0	B2	0,088	0,102	0,152	0,306	0,451	0,507
		Average	0,087	0,099	0,149	0,293	0,429	0,481
	1	B5	0,088	0,097	0,144	0,292	0,459	0,566
	1	B6	0,086	0,094	0,135	0,243	0,376	0,442
		Average	0,087	0,096	0,139	0,267	0,417	0,504
fluorescence	0	Mean	401	361	297	314	336	329
butyric acid	0	Mean	394	361	296	315	342	340
		Average	397,5	361	296,5	314,5	339	334,5
Relative Fluorescence	0		4585	3634	1991	1074	790	696
	1	Mean	414	354	332	318	333	386
	1	Mean	403	381	304	324	339	350
		Average	408,5	367,5	318	321	336	368
Relative Fluorescence	1		4690	3844	2286	1201	805	730
x-fold induction	1 mM		1,0	1,1	1,1	1,1	1,0	1,0

measurement 1	mM	t in h	0	4	8	12	16	20
absorbance	0	C1	0,089	0,101	0,161	0,318	0,429	0,477
iso-butyric acid	0	C2	0,092	0,103	0,159	0,354	0,485	0,540
		Average	0,091	0,102	0,160	0,336	0,457	0,508
	0,5	C3	0,094	0,107	0,169	0,382	0,541	0,604
	0,5	C4	0,092	0,104	0,164	0,357	0,510	0,575
		Average	0,093	0,105	0,167	0,370	0,526	0,590
fluorescence	0	Mean	910	316	268	239	241	250
iso-butyric acid	0	Mean	937	300	235	241	246	253
		Average	923,5	308	251,5	240	243,5	251,5
Relative Fluorescence	0		10199	3017	1571	714	533	495
	0,5	Mean	930	298	235	238	242	244
	0,5	Mean	893	320	237	254	266	272
		Average	911,5	309	236	246	254	258
Relative Fluorescence	0,5		9801	2930	1417	666	483	438
x-fold induction	0,5 mM		1,0	1,0	0,9	0,9	0,9	0,9

measurement 2	mM	t in h	0	4	8	12	16	20
absorbance	0	C1	0,092	0,103	0,160	0,322	0,439	0,489
iso-butyric acid	0	C2	0,095	0,104	0,163	0,345	0,481	0,547
		Average	0,093	0,103	0,161	0,333	0,460	0,518
	0,5	C3	0,094	0,102	0,161	0,345	0,490	0,556
	0,5	C4	0,085	0,097	0,150	0,317	0,450	0,504
		Average	0,089	0,100	0,156	0,331	0,470	0,530
fluorescence	0	Mean	372	354	287	315	204	218
iso-butyric acid	0	Mean	353	343	288	322	203	204
		Average	362,5	348,5	287,5	318,5	203,5	211
Relative Fluorescence	0		3885	3379	1781	955	442	407
	0,5	Mean	371	343	281	295	191	200
	0,5	Mean	362	346	275	292	187	190
		Average	366,5	344,5	278	293,5	189	195
Relative Fluorescence	0,5		4097	3450	1788	886	402	368
x-fold induction	0,5 mM		1,1	1,0	1,0	0,9	0,9	0,9

measurement 3		t in h	0	4	8	12	16	20
absorbance	IBA 0	C1	0,087	0,093	0,137	0,266	0,394	0,444
iso-butyric acid	IBA 0	C2	0,092	0,095	0,136	0,280	0,417	0,471
		Average	0,089	0,094	0,137	0,273	0,406	0,457
	IBA 0,5	C3	0,099	0,121	0,182	0,393	0,612	0,618
	IBA 0,5	C4	0,092	0,102	0,153	0,341	0,541	0,597
		Average	0,095	0,111	0,167	0,367	0,576	0,608
fluorescence	IBA 0	Mean	400	367	297	320	332	343
iso-butyric acid	IBA 0	Mean	376	366	297	320	327	338
		Average	388	366,5	297	320	329,5	340,5
Relative Fluorescence	0		4340	3889	2175	1173	812	745
	IBA 0,5	Mean	381	356	292	306	328	327
	IBA 0,5	Mean	414	371	325	324	347	341
		Average	397,5	363,5	308,5	315	337,5	334
Relative Fluorescence	0,5		4167	3264	1842	858	586	550
x-fold induction	0,5 mM		1,0	0,8	0,8	0,7	0,7	0,7

measurement 1	mM	t in h	0	4	8	12	16	20
absorbance	0	D1	0,086	0,102	0,173	0,330	0,451	0,501
valeric acid	0	D2	0,081	0,095	0,144	0,295	0,412	0,459
		Average	0,084	0,099	0,159	0,313	0,431	0,480
	0,5	D3	0,080	0,104	0,157	0,318	0,504	0,558
	0,5	D4	0,096	0,129	0,221	0,509	0,620	0,608
		Average	0,088	0,117	0,189	0,413	0,562	0,583
fluorescence	0	Mean	920	290	225	235	241	260
valeric acid	0	Mean	943	300	232	236	238	260
		Average	931,5	295	228,5	235,5	239,5	260
Relative Fluorescence	0		11142	2983	1441	754	555	541
	0,5	Mean	930	296	247	270	279	308
	0,5	Mean	980	299	259	303	319	335
		Average	955	297,5	253	286,5	299	321,5
Relative Fluorescence	0,5		10828	2553	1336	693	532	552
x-fold induction	0,5 mM		1,0	0,9	0,9	0,9	1,0	1,0

measurement 2	mM	t in h	0	4	8	12	16	20
absorbance	0	D1	0,086	0,100	0,159	0,306	0,431	0,482
valeric acid	0	D2	0,088	0,103	0,162	0,328	0,447	0,491
		Average	0,087	0,102	0,160	0,317	0,439	0,487
	0,5	D3	0,090	0,104	0,167	0,362	0,572	0,545
	0,5	D4	0,087	0,100	0,154	0,307	0,459	0,564
		Average	0,089	0,102	0,161	0,334	0,515	0,555
fluorescence	0	Mean	360	331	276	303	198	209
valeric acid	0	Mean	357	340	284	315	205	217
		Average	358,5	335,5	280	309	201,5	213
Relative Fluorescence	0		4125	3302	1746	973	459	438
	0.5	Mean	369	340	297	349	261	264
	0.5	Mean	353	331	290	318	246	284
		Average	361	335,5	293,5	333,5	253,5	274
Relative Fluorescence	0,5		4063	3292	1828	998	492	494
x-fold induction	0,5 mM		1,0	1,0	1,0	1,0	1,1	1,1

measurement 3	mM	t in h	0	4	8	12	16	20
absorbance	0	D1	0,088	0,095	0,139	0,269	0,399	0,449
valeric acid	0	D2	0,088	0,096	0,146	0,298	0,436	0,491
		Average	0,088	0,095	0,143	0,283	0,418	0,470
	0,5	D3	0,082	0,097	0,147	0,288	0,454	0,552
	0,5	D4	0,091	0,131	0,202	0,389	0,544	0,491
		Average	0,086	0,114	0,175	0,339	0,499	0,522
fluorescence	0	Mean	380	346	282	292	321	334
valeric acid	0	Mean	393	353	311	306	323	324
		Average	386,5	349,5	296,5	299	322	329
Relative Fluorescence	0		4405	3673	2078	1055	771	699
	0.5	Mean	393	347	310	323	390	455
	0.5	Mean	381	368	327	329	355	376
		Average	387	357,5	318,5	326	372,5	415,5
Relative Fluorescence	0,5		4477	3136	1825	962	747	797
x-fold induction	0,5 mM		1,0	0,9	0,9	0,9	1,0	1,1

measurement 1	mM	t in h	0	4	8	12	16	20
absorbance	0	E1	0,091	0,097	0,150	0,302	0,419	0,468
iso-valeric acid	0	E2	0,089	0,104	0,151	0,323	0,444	0,494
		Average	0,090	0,100	0,150	0,313	0,431	0,481
	0,5	E3	0,102	0,140	0,214	0,439	0,625	0,717
	0,5	E4	0,093	0,116	0,197	0,470	0,643	0,631
		Average	0,098	0,128	0,206	0,455	0,634	0,674
fluorescence	0	Mean	930	295	236	231	239	249
iso-valeric acid	0	Mean	960	296	234	236	246	265
		Average	945	295,5	235	233,5	242,5	257
Relative Fluorescence	0		10518	2940	1564	747	562	534
	0,5	Mean	936	287	233	235	251	280
	0,5	Mean	928	319	256	233	239	253
		Average	932	303	244,5	234	245	266,5
Relative Fluorescence	0,5		9554	2363	1189	515	386	395
x-fold induction	0,5 mM		0,9	0,8	0,8	0,7	0,7	0,7

measurement 2	mM	t in h	0	4	8	12	16	20
absorbance	0	E1	0,085	0,102	0,155	0,316	0,436	0,485
iso-valeric acid	0	E2	0,091	0,100	0,153	0,325	0,445	0,495
		Average	0,088	0,101	0,154	0,320	0,441	0,490
	0,5	E3	0,093	0,114	0,186	0,417	0,737	0,400
	0,5	E4	0,089	0,099	0,154	0,336	0,434	0,428
		Average	0,091	0,106	0,170	0,376	0,585	0,414
fluorescence	0	Mean	362	334	283	300	205	220
iso-valeric acid	0	Mean	349	358	283	295	199	203
		Average	355,5	346	283	297,5	202	211,5
Relative Fluorescence	0		4051	3431	1836	929	459	432
	0.5	Mean	347	337	280	308	198	219
	0.5	Mean	344	338	286	312	189	198
		Average	345,5	337,5	283	310	193,5	208,5
Relative Fluorescence	0,5		3809	3173	1668	823	331	504
x-fold induction	0,5 mM		0,9	0,9	0,9	0,9	0,7	1,2

measurement 3	mM	t in h	0	4	8	12	16	20
absorbance	0	E1	0,087	0,095	0,137	0,269	0,395	0,444
iso-valeric acid	0	E2	0,095	0,105	0,160	0,342	0,490	0,545
		Average	0,091	0,100	0,148	0,305	0,442	0,495
	0,5	E3	0,090	0,104	0,159	0,340	0,496	0,557
	0,5	E4	0,099	0,130	0,194	0,421	0,654	0,730
		Average	0,094	0,117	0,176	0,381	0,575	0,644
fluorescence	0	Mean	389	338	288	290	333	336
iso-valeric acid	0	Mean	395	342	292	305	327	326
		Average	392	340	290	297,5	330	331
Relative Fluorescence	0		4303	3402	1954	974	746	669
	0.5	Mean	389	350	303	319	329	336
	0.5	Mean	399	360	313	338	359	361
		Average	394	355	308	328,5	344	348,5
Relative Fluorescence	0,5		4183	3041	1747	863	598	541
x-fold induction	0,5 mM		1,0	0,9	0,9	0,9	0,8	0,8

FL: Fluorescence
Abs: Absorbance
MM: Minimal Medium
AA: Acetic Acid

Row Data Figure 5B

	measurement	t in h	0	2	4	6	8	10
**integrated**	1	absorbance	0,228	0,247	0,273	0,307	0,349	0,393
15 mM acetic acid		fluorescence	103	110	140	178	221	274
		**relative fluorescence**	**451**	**445**	**512**	**581**	**632**	**698**
	2	absorbance	0,232	0,255	0,290	0,326	0,360	0,368
		fluorescence	104	119	168	216	265	304
		**relative fluorescence**	**447**	**468**	**580**	**662**	**735**	**825**
	3	absorbance	0,240	0,248	0,272	0,285	0,298	0,294
		fluorescence	135	144	157	179	198	208
		**relative fluorescence**	**564**	**580**	**577**	**627**	**663**	**708**

	measurement	t in h	0	2	4	6	8	10
**plasmid-based**	1	absorbance	0,253	0,272	0,342	0,465	0,603	0,670
15 mM acetic acid		fluorescence	1332	1547	2051	3363	5734	8772
		**relative fluorescence**	**5272**	**5687**	**5992**	**7231**	**9508**	**13085**
	2	absorbance	0,250	0,244	0,314	0,453	0,602	0,672
		fluorescence	1070	1130	1802	3365	6190	9956
		**relative fluorescence**	**4278**	**4624**	**5735**	**7435**	**10274**	**14812**
	3	absorbance	0,233	0,258	0,331	0,471	0,590	0,663
		fluorescence	708	888	1586	3017	5394	8520
		**relative fluorescence**	**3035**	**3439**	**4796**	**6409**	**9143**	**12853**

	measurement	t in h	0	2	4	6	8	10
**S:R = 1:10**	1	absorbance	0,248	0,265	0,314	0,381	0,451	0,483
15 mM acetic acid		fluorescence	62	71	322	2314	6952	12589
		**relative fluorescence**	**250**	**266**	**1024**	**6080**	**15404**	**26086**
	2	absorbance	0,259	0,268	0,325	0,409	0,477	0,513
		fluorescence	64	79	559	3293	8192	13904
		**relative fluorescence**	**247**	**295**	**1722**	**8053**	**17168**	**27079**
	3	absorbance	0,249	0,276	0,326	0,394	0,444	0,493
		fluorescence	108	116	701	3992	9530	15759
		**relative fluorescence**	**436**	**419**	**2151**	**10129**	**21479**	**31978**

	measurement	t in h	0	2	4	6	8	10
**PC:R = 1:10**	1	absorbance	0,251	0,282	0,373	0,464	0,523	0,572
0 mM acetic acid		fluorescence	64	213	2914	10048	18463	26310
		**relative fluorescence**	**255**	**756**	**7802**	**21640**	**35297**	**45984**
	2	absorbance	0,261	0,290	0,385	0,473	0,534	0,583
		fluorescence	107	308	3131	10029	17881	24805
		**relative fluorescence**	**411**	**1062**	**8135**	**21193**	**33481**	**42517**
	3	absorbance	0,245	0,291	0,385	0,485	0,551	0,591
		fluorescence	45	138	2154	8690	15990	23174
		**relative fluorescence**	**184**	**473**	**5598**	**17929**	**29015**	**39240**
